# Targeting histone deacetylase 3 (HDAC3) in the bone marrow microenvironment inhibits multiple myeloma proliferation by modulating exosomes and IL-6 trans-signaling

**DOI:** 10.1038/s41375-019-0493-x

**Published:** 2019-05-29

**Authors:** Matthew Ho, Tianzeng Chen, Jiye Liu, Paul Dowling, Teru Hideshima, Li Zhang, Eugenio Morelli, Gulden Camci-Unal, Xinchen Wu, Yu-Tzu Tai, Kenneth Wen, Mehmet Samur, Robert L. Schlossman, Ralph Mazitschek, Emma L. Kavanagh, Sinéad Lindsay, Takeshi Harada, Amanda McCann, Kenneth C. Anderson, Peter O’Gorman, Giada Bianchi

**Affiliations:** 1 LeBow Institute for Myeloma Therapeutics and Jerome Lipper Multiple Myeloma Center, Department of Medical Oncology, Dana Farber Cancer Institute, Harvard Medical School, Boston, MA 02115 USA; 2 UCD Conway Institute of Biomolecular and Biomedical Science, UCD School of Medicine, University College Dublin, Belfield (UCD), Dublin 4, Ireland; 3 Biology Department, National University of Ireland Maynooth, Co. Kildare, Kildare, Ireland; 4 Department of Hematology, West China Hospital, Sichuan University, Chengdu, Sichuan China; 5 Department of Chemical Engineering, University of Massachusetts Lowell, One University Avenue, Lowell, MA 01854 USA; 6 Biomedical Engineering and Biotechnology Program, University of Massachusetts Lowell, One University Avenue, Lowell, MA 01854 USA; 7 Center for Systems Biology, Massachusetts General Hospital, Boston, MA USA; 8 Department of Medicine and Bioregulatory Sciences, University of Tokushima Graduate School of Medicine, 3-18-15 Kuramoto, Tokushima, 770-8503 Japan; 9 Haematology Department, Mater Misericordiae University Hospital, Dublin, Ireland

**Keywords:** Translational research, Myeloma

## Abstract

Multiple myeloma (MM) is an incurable cancer that derives pro-survival/proliferative signals from the bone marrow (BM) niche. Novel agents targeting not only cancer cells, but also the BM-niche have shown the greatest activity in MM. Histone deacetylases (HDACs) are therapeutic targets in MM and we previously showed that HDAC3 inhibition decreases MM proliferation both alone and in co-culture with bone marrow stromal cells (BMSC). In this study, we investigate the effects of HDAC3 targeting in BMSCs. Using both BMSC lines as well as patient-derived BMSCs, we show that HDAC3 expression in BMSCs can be induced by co-culture with MM cells. Knock-out (KO), knock-down (KD), and pharmacologic inhibition of HDAC3 in BMSCs results in decreased MM cell proliferation; including in autologous cultures of patient MM cells with BMSCs. We identified both quantitative and qualitative changes in exosomes and exosomal miRNA, as well as inhibition of IL-6 trans-signaling, as molecular mechanisms mediating anti-MM activity. Furthermore, we show that HDAC3-KD in BM endothelial cells decreases neoangiogenesis, consistent with a broad effect of HDAC3 targeting in the BM-niche. Our results therefore support the clinical development of HDAC3 inhibitors based not only on their direct anti-MM effects, but also their modulation of the BM microenvironment.

## Introduction

Multiple myeloma (MM) is a cancer of terminally differentiated plasma cells that accounts for 1.3% of all malignancies and 15% of hematological cancers, making it the second most common blood cancer after non-Hodgkin lymphoma [1]. Despite significant therapeutic advances with high dose therapy and stem cell transplantation as well as novel therapies, relapses occur in most patients and MM remains incurable. We and others have shown that the bone marrow (BM) microenvironment supports MM proliferation and survival, as well as confers cell adhesion mediated-drug resistance (CAM-DR) [2]. Our increasing understanding of MM biology has led to the discovery of novel therapeutic targets such as histone deacetylases (HDACs) in both tumor cells and BM milieu, and the pan-HDAC inhibitor panobinostat has been FDA approved to treat relapsed, refractory MM [3, 4]. However, clinical trials have shown that nonselective HDAC inhibitors have narrow therapeutic index, prompting the development of isoform-specific HDAC inhibitors such as the HDAC6 inhibitor ricolinostat, currently in clinical trials [5].

We previously showed that HDAC3-knockdown (KD) or HDAC3-selective inhibition with BG45 attenuates MM proliferation, both alone and in co-culture with bone marrow stromal cells (BMSCs) [6]. There is a growing interest in understanding the effects of novel drugs on the BM niche as it has become increasingly evident that drugs targeting both cancer cells and their microenvironment have been associated with higher therapeutic success [7, 8]. In this study, we investigated the effects of targeting HDAC3 in BMSCs and BM endothelial cells (BMEC) on MM pathogenesis. We show that HDAC3 knock-down (KD) and knock-out (KO) in both human BM stromal cell lines and primary BMSCs derived from patients with newly diagnosed (NDMM) and refractory relapsed MM (RRMM) significantly decreased BMSC-induced MM cell proliferation and survival in vitro and in vivo. We further show that HDAC3 KD in BMSCs inhibits cell adhesion mediated-drug resistance (CAM-DR) to doxorubicin, and that HDAC3 KD in BMECs leads to a significant inhibition of neo-angiogenesis. We studied the molecular mechanisms mediating these phenotypes and discovered that HDAC3 KD in BMSC causes increased secretion of soluble glycoprotein 130 (sgp130), which results in dysregulation of IL-6 trans-signaling, a well-established pro-survival pathway in MM. By using proteomics and micro RNA (miRNA) sequencing, we detected changes in exosomes derived from HDAC3 KD versus scramble control co-culture systems [9]. Altogether, our study supports the clinical development of HDAC3 inhibitors based not only on their direct, anti-MM effect, but also on their indirect effects on the BM milieu, resulting in decreased MM cell growth and survival.

## Materials and methods

### siRNA transfection and HDAC3 overexpression

siRNAs against HDAC3 were purchased from Dharmacon™ (GE Healthcare Life Sciences, Marlborough, MA, USA). For siRNA transfection of BMSCs, Lipofectamine RNAiMax (Thermo Fisher Scientific, Waltham, MA, USA) was used. For siRNA transfection of CD138^−^ BMMCs, the NEON® transfection system (Thermo Fisher Scientific) was used.

In order to overexpress HDAC3, HS-5 cells were transiently transfected with HDAC3-Flag tagged protein using Lipofectamine 2000 (Thermo Fisher Scientific) according to the manufacturer’s protocol. HDAC3-Flag was a gift from Eric Verdin (Addgene plasmid # 13819).

**Table:** 

ON-TARGETplus non-targeting control			
siRNA #	Catalog number	Target sequence	Abbreviated as
1	D-001810-01-05	NA	Scramble
ON-TARGETplus human HDAC3			
siRNA #	Catalog number	Target sequence	Abbreviated as
1	J-003496-09	AAAGCGAUGUGGAGAUUUA	HDAC3 #1
3	J-003496-11	GGAAUGCGUUGAAUAUGUC	HDAC3 #3

### CRISPR-cas9 knockout of HDAC3

We designed single guide RNA (sgRNA) targeting hsHDAC3 by using MIT CRISPR tool. HDAC3 sgRNA was then cloned into pSpCas9(BB)-2A-GFP vector, as previously reported [10]. Positive monoclones were screened via western blot based on absent HDAC3 expression. Bi-allelic KO was confirmed via genomic PCR and TA-cloning of target gene locus. The following sgRNA against exon 1 of HDAC3 was used: TATTTCTACGACCCCGACGT. pSpCas9(BB)-2A-GFP (PX458) was a gift from Feng Zhang (Addgene plasmid # 48138).

### Luciferase proliferation assay

BMSC were transfected with HDAC3 siRNA, HDAC3 overexpression vector or treated with BG45 for the indicated times. After 48–96h, media was removed and cells washed prior to adding MM1S.Luc or H929.Luc for a further 48–96h. Proliferation was measured using the Luciferase assay according to manufacturer’s protocol (Promega, Madison, WI, USA).

### Murine xenograft models

Five-week-old female severe combined immunodeficiency-beige (SCID-beige) mice were used for this study. All animal studies were performed under a protocol approved by the Animal Ethics Committee of the DFCI. Mice were subcutaneously injected with a co-culture of 5 × 10^6^ viable MM1S.Luc and 5 × 10^6^ viable HS-5 (HDAC3 WT or HDAC3 KO) cells in a 1:1 ratio with Matrigel™ (Invitrogen, Carlsbad, CA, USA). MM1S.Luc tumor burden was measured weekly using whole-body bioluminescence imaging (BLI). Mice were sacrificed when the tumor reached 2 cm in length or 2 cm^3^ volume or if mice appeared moribund, to prevent unnecessary morbidity.

### Statistical analysis

Experiments were performed at least three times unless otherwise specified. Biological triplicates were used unless otherwise specified. Statistical significance was determined by Student’s *t*-test after determination of normal distribution with *F*-test. (NS: *P* > 0.05; *P ≤ 0.05; **P ≤ 0.01; ***P ≤ 0.001; ****P ≤ 0.0001).

## Results

### BMSC derived from MM patients have increased expression of HDAC3 which results in enhanced MM cell proliferation

**Fig. 1:**
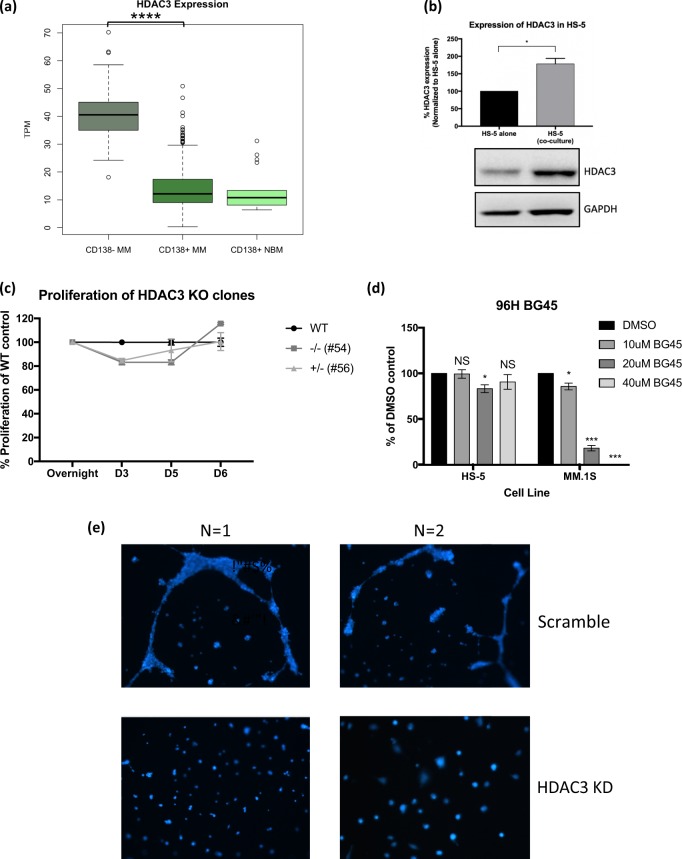
HDAC3 expression is increased in MM-derived BMSC compared to healthy donor-derived BMSC but HDAC3 is not necessary for BMSC viability or proliferation. **a** Gene expression analysis from the IFM/DFCI 2009 dataset revealed that HDAC3 expression is higher in CD138− BMMCs when compared to CD138+ BMMCs derived from patients with MM. **b** Co-culture of HS-5 cells with MM1S.Luc triggers induction of HDAC3 expression in BMSC. GAPDH was used as loading control. Quantification was performed using ImageJ. **c** HDAC3 KO HS-5 cells have comparable viability to HDAC3 WT HS-5 cells as assessed by CCK-8 assay. **d** HDAC3 inhibition using BG45 is not cytotoxic towards HS-5 even at doses two-folds higher than the Ec50 of MM1S as measured by CCK-8 assay at 96 h. **e** Endothelial tube formation assay shows compromised formation of endothelial tubes in BMEC60 cells transfected with HDAC3 siRNA compared to scrambled siRNA. Two representative, independent experiments shown

First, we sought to determine the relative expression of HDAC3 in the CD138− BMMC fraction versus the CD138+ (plasma cell) BMMC fraction. Gene expression analysis from the IFM/DFCI 2009 dataset showed that CD138− BMMCs had higher expression of HDAC3 compared to CD138+ BMMCs (Fig. 1a). Consistent with this data, western blot analysis showed that HDAC3 is more highly expressed in the HS-5 bone marrow stromal cell line compared to MM.1S MM cell line (Fig. S1a), leading us to hypothesize that targeting HDAC3 in stroma could potentially have a profound impact on the MM survival and proliferation in the context of the BM microenvironment.

Next, to investigate whether co-culture of MM and BMSC would lead to changes in expression of HDAC3 in either cell type, we cultured luciferase expressing MM.1S cells (MM1S.Luc) on a monolayer of HS-5 BMSC line, followed by magnetic bead selection for CD138+ MM cells. Our data show that co-culture induces increased HDAC3 expression in HS-5 BMSCs, but not in MM1S.Luc cells (Figs. 1b, S1b). Consistent with this data, gene expression profiling show that co-culture with HS-5 does not alter HDAC3 expression in a panel of MM cell lines (Fig. S1c). Importantly, BMSC derived from MM patients (MM-BMSC) express higher levels of HDAC3, as assessed by western blotting, compared to healthy donor BMSC (HD-BMSC) (Fig. S1d), further corroborating the in vitro finding that HDAC3 expression in BMSCs can be induced by MM cells.

To functionally assess whether increased HDAC3 expression in BMSC results in enhanced MM proliferation/viability, we transiently overexpressed FLAG-tagged HDAC3 (HDAC3-OE) in HS-5 BMSCs. MM1S.Luc cells proliferation was increased in co-culture with HDAC3 OE HS-5 BMSCs compared to cultures with HS-5 transfected with an empty, backbone vector (BB) (28% increase in MM proliferation; *P* value < 0.05) (Fig. S2a, S2b).

### HDAC3 is not essential for BMSC and BMEC survival or proliferation, but HDAC3 KD increases MM to BMSC chemotaxis and inhibits neo-angiogenesis

Next, we asked whether HDAC3 expression is necessary for BMSC survival. siRNA-mediated HDAC3 KD as well as monoallelic (clone #56) and biallelic (clone #54) HDAC3 KO show that HDAC3 does not impact BMSC viability (Figs. S3a–d,  1c, S4). Similarly, pharmacological inhibition of HDAC3 using the HDAC3-selective inhibitor BG45 does not trigger significant BMSC growth inhibition, even at concentrations up to two-fold higher than the EC_50_ for MM.1S cells (Figs. 1d, S3e). However, HDAC3 KD in HS-5 BMSCs triggered increased MM chemotaxis (Fig. S5a). Based on our cytokine profiling data, we hypothesized that this phenotype was mediated by increased CXCL1 (GRO-alpha) (Fig. S5b). To test this hypothesis, we used anti-CXCL1 neutralizing antibody (15 µg/ml) in migration assays and show that it abrogates MM transmigration towards HDAC3 KD HS-5 cells (Fig. S5a).

Similar to BMSCs, HDAC3 silencing in BMECs only modestly decreases their viability (Fig. S6). However, HDAC3 KD significantly inhibits endothelial tube formation, indicating that HDAC3 function in BMECs is necessary for adequate neo-angiogenesis (Fig. 1e) [11].

### Targeting HDAC3 in BMSC decreases BMSC-induced MM cell line and primary MM cell proliferation

**Fig. 2:**
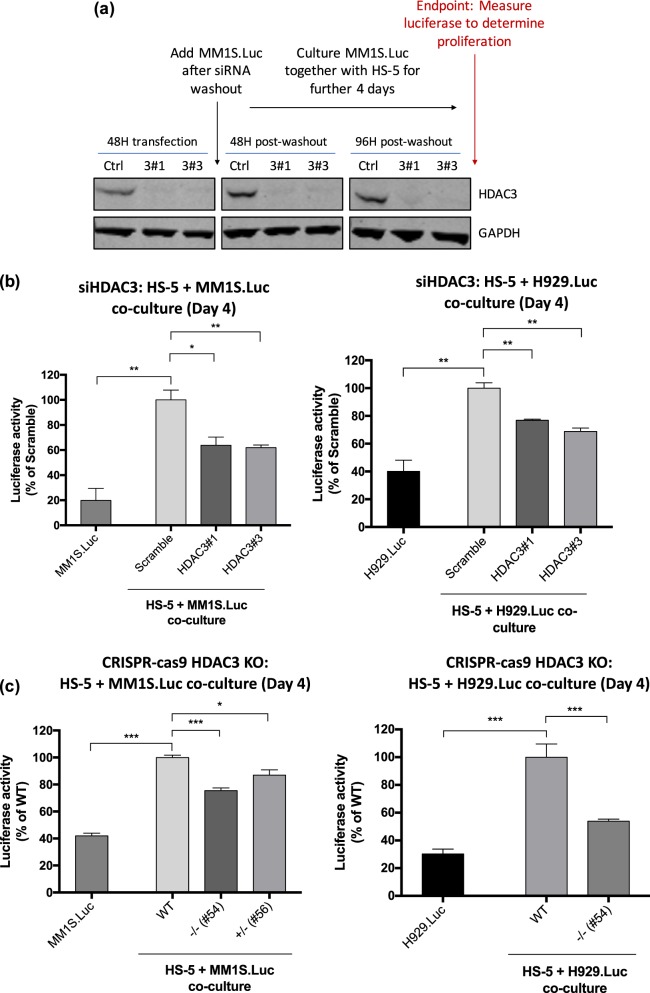
HDAC3 knockdown (KD) and knockout (KO) in BMSCs triggers significant MM cell growth inhibition in MM-BMSC co-culture setting. **a** Co-culture experiment schema and western blot showing HDAC3 siRNA knockdown in HS-5 cells after 48 h of transfection. HDAC3 was silenced in HS-5 BMSCs using siRNA for 48 h. The transfection mix was subsequently washed out and MM1S.Luc/H929.Luc was added in co-culture for a further 4 days before luciferase was performed to measure MM proliferation. The HDAC3 KD in HS-5 cells persists up to 96 h after transfection mix is washed out. GAPDH is used as a loading control. **b** HDAC3 siRNA knockdown in HS-5 significantly inhibits MM1S.Luc proliferation as measured by Luciferase Assay (left chart: 37.1% mean decrease in MM1S.Luc proliferation when cocultured with HDAC3 KD HS-5, *P* < 0.05). HDAC3 siRNA KD in HS-5 significantly inhibits H929.Luc proliferation as measured by Luciferase Assay (right chart: 27.2% mean decrease in H929.Luc proliferation when cocultured with HDAC3 KD HS-5, *P* < 0.05). **c** Left chart: Homozygous HDAC3 KO HS-5 clone #54 inhibited the proliferation of MM1S.Luc to a greater extent than heterozygous HDAC3 KO HS-5 clone #56 when compared to HDAC3 WT HS-5 clone. Right chart: Homozygous HDAC3 KO HS-5 clone #54 inhibited the proliferation of H929.Luc compared to HDAC3 WT HS-5 clone

To evaluate the effect of HDAC3-silencing in HS-5 BMSCs on MM proliferation, we co-cultured MM1S.Luc and H929.Luc MM cells for 4 days with HS-5 BMSCs previously transfected with HDAC3 siRNA or scrambled siRNA and assessed MM cell proliferation using luciferase assay (Fig. 2a). HDAC3 KD significantly inhibits MM1S.Luc and H929.Luc MM cell proliferation (36.1% and 27.2% mean decrease, respectively, *P* value < 0.05) (Figs. 2b, S7a). A similar pattern of reduction in MM proliferation is observed when HDAC3 KD was performed in MSP-1 cells, a MM-BMSC-derived cell line (14% decrease, *P* value < 0.05) (Fig. S7b). To assess whether KD of other HDAC class I members results in similar anti-proliferative effects, we performed HDAC1 and HDAC2 KD in HS-5 BMSCs prior to co-culture with MM cell lines. Our results show that HDAC1 KD had no effect on MM proliferation, while targeting HDAC2 increases MM proliferation (Fig. S8a, S8b). As an alternative strategy to target HDAC3, we also used HDAC3 monoallelic and biallelic KO HS-5 BMSC clones. Significant reduction in MM1S.Luc proliferation was noted in co-cultures with these mono and bi-allelic KO HS-5 cells compared to co-culture with HDAC3 WT HS-5 cells (Fig. 2c). A significant reduction in H929.Luc proliferation was also observed in co-culture with bi-allelic KO HS-5 cells (Figs. 2c, S7c).

Pharmacological inhibition of HDAC3 (Fig. S8c) in HS-5 BMSCs via BG45 decreases MM proliferation when compared to MM cells cultured with DMSO-treated HS-5 cells (18% decrease, *P* value < 0.05) (Fig. S9a). We confirmed that HDAC3 inhibition persisted during these co-cultures based upon expression of acetylated-H3K9 (Fig. S9b).

**Fig. 3:**
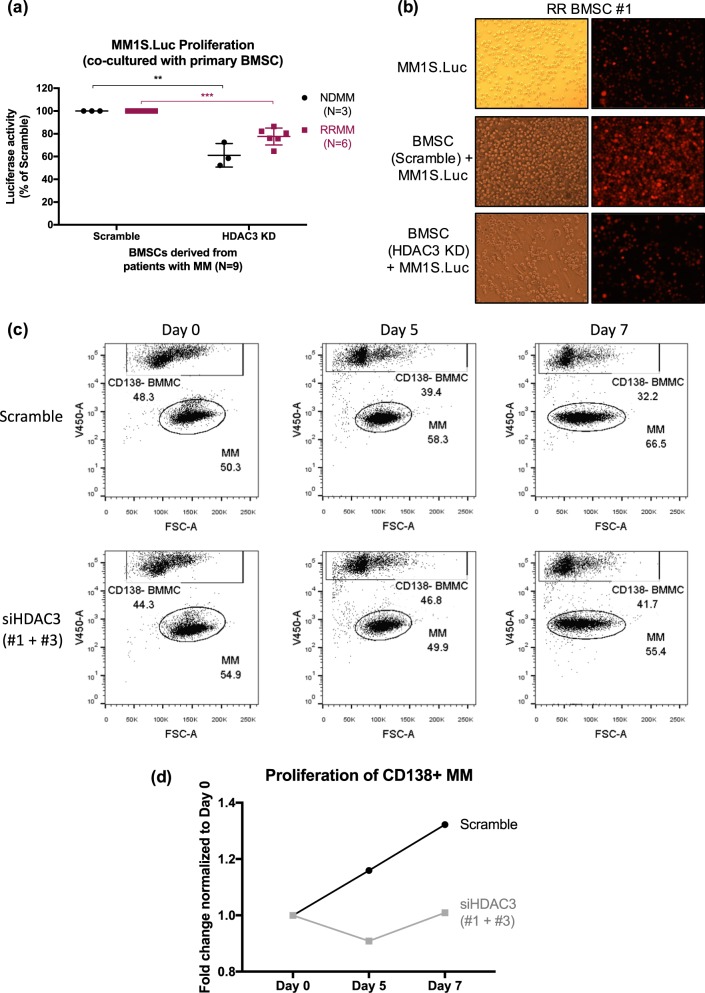
HDAC3 knockdown (KD) in primary human BM stromal cells derived from newly-diagnosed MM (ND BMSC), and refractory-relapsed MM (RR BMSC) trigger significant MM cell growth inhibition in MM-MSC co-culture setting. **a** Figure comparing MM1S.Luc proliferation in co-culture with scramble (left) or HDAC3 si-RNA transfected (right) newly diagnosed (ND, black dots) and refractory relapsed (RR, red squares) BMSC respectively. **b** Light microscopy images (left) showing the co-culture of MM1S.Luc with scramble or HDAC3 KD BMSCs and fluorescent microscopy images (right) showing mCherry-labeled MM1S.Luc cells co-cultured with scramble or HDAC3 KD BMSCs derived from RR MM (RR BMSC #1). **c** Dot plot panels show the relative proportion of CD138 negative bone marrow mononuclear cells (BMMC, top rectangle) and CD138 positive MM cells (lower oval) over indicated times in an autologous co-culture system of primary cells obtained from a patient with RRMM. Top panels represent scrambled-transfected CD138 negative, bone marrow mononuclear cells (BMMC), lower panels show HDAC3 siRNA-transfected BMMC. **d** Chart representing fold changes in MM cell proportion over indicated times in scrambled-transfected and HDAC3 siRNA-transfected BMMC co-culture

Moreover, HDAC3 KD in MM-BMSC obtained from patients with newly-diagnosed MM (NDMM; *N* = 3) and refractory-relapsed MM (RRMM; *N* = 6) also significantly decreases BMSC-induced MM1S.Luc proliferation in the co-culture system (Figs. 3a, b,  S10a, S10b). As a negative control we used healthy donor-derived BMSCs (HD-BMSC), which do not express HDAC3 (Fig. S1d, S10c). Consistently, HDAC3 KD in HD-BMSC does not affect MM1S.Luc cell proliferation in the co-culture system (Fig. S10c). Importantly, we demonstrate that HDAC3 KD (but not scrambled siRNA KD) in primary CD138− BMMCs significantly decreases proliferation of autologous CD138+ MM cells derived from a patient with RRMM (Figs. 3c, S11a–c).

### Paracrine-autocrine loop between HDAC3 KD BMSC and MM cells leads to inhibition of IL-6 trans-signaling

**Fig. 4:**
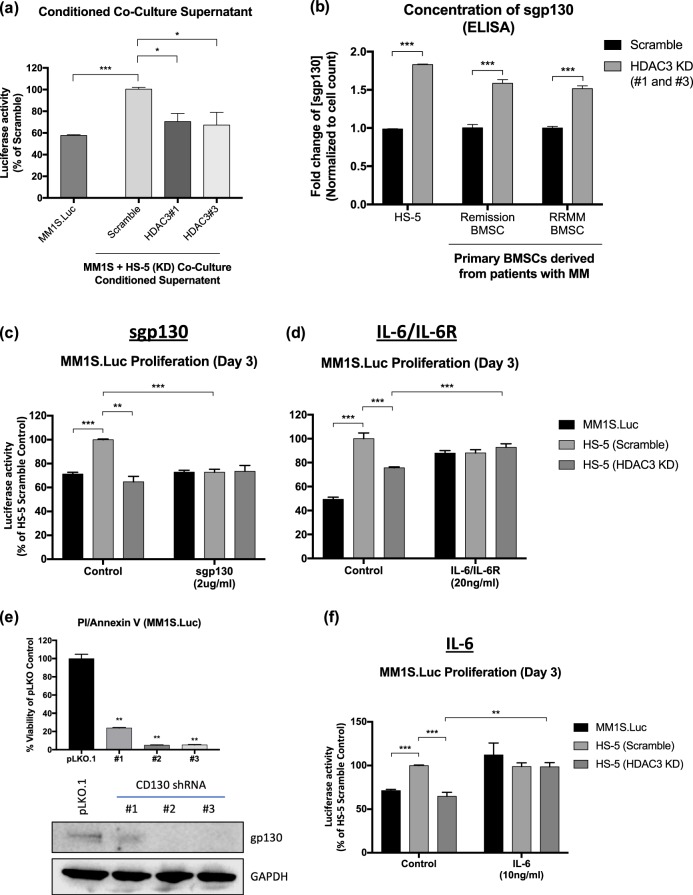
Conditioned supernatant from HDAC3 KD HS-5 plus MM1S.Luc co-culture triggers significant MM cell growth inhibition through the attenuation of IL-6 trans-signaling. **a** Conditioned media (CM) from HDAC3 KD HS-5 and MM1S.Luc co-culture inhibits MM1S.Luc proliferation when compared to CM from the co-culture of scrambled HS-5 and MM1S.Luc (31.2% mean decrease in MM1S.Luc proliferation in HDAC3 KD, *P* < 0.05). **b** ELISA for soluble-gp130 (sgp130) shows increase in sgp130 in the co-culture supernatant of HDAC3-silenced HS-5 (1.8-fold), and HDAC3-silenced primary BMSCs derived from a patient with MM in partial remission (1.6-fold) and RRMM (1.5-fold), respectively. **c** Treatment with 2 µg/ml of exogenous human recombinant sgp130 abrogates HS-5 induced MM1S.Luc proliferation while **d** treatment with 20 ng/ml of human recombinant IL6/IL6R chimera rescues MM1S.Luc growth inhibition induced by HDAC3-silencing in HS-5. **e** Silencing of CD130 in MM1S.Luc results in significant cell death as measured by PI/Annexin V apoptosis assay. Western blot showing CD130 KD via three independent shRNAs. GAPDH was used as loading control. **f** IL-6 alone was also able to rescue MM1S.Luc growth inhibition induced by HDAC3-silencing in HS-5

Next, we asked whether the observed anti-MM proliferative effect of HDAC3 KD/KO in BMSC was due to impaired MM-BMSC adhesion. However, adhesion assays showed no significant changes in MM-BMSC adhesion between scrambled vs HDAC3 KD HS-5 cells (Fig. S12a, S12b). Having excluded diminished cell-to-cell contact as a potential molecular mechanism, we next investigated autocrine/paracrine factors that could impact MM proliferation and viability in co-cultures. To this end, we harvested conditioned media (CM) from the co-cultures of HDAC3 KD versus scrambled siRNA HS-5 BMSCs with MM1S.Luc cells and used it as culture medium for MM1S.Luc alone. CM obtained from HDAC3 KD HS-5 and MM1S.Luc co-cultures significantly inhibited MM1S.Luc proliferation (31.2% mean decrease, *P* value < 0.05) compared to CM obtained from scrambled siRNA KD HS5 co-cultured with MM1S.Luc (Fig. 4a). Consistent with this, CM obtained from the co-culture of HDAC3 KD HS-5 and H929.Luc significantly inhibited H929.Luc proliferation (Fig. S13a) and CM obtained from the co-culture of HDAC3 KD NDMM-derived BMSC and MM1S.Luc significantly inhibited MM1S.Luc proliferation (Fig. S13b). Interestingly, CM from HDAC3 KD HS-5 or NDMM-derived BMSC cells alone failed to show an anti-proliferative effect, suggesting paracrine-autocrine loop between HDAC3 KD BMSC and MM cells alters the secretome in a way that decreases the proliferative advantage induced by the microenvironment (Fig. S13c,  S13d). Cytokine profiling showed a 1.8-fold increase in soluble glycoprotein 130 (sgp130), a natural inhibitor of the IL6-transignaling pathway, in co-culture supernatants from HDAC3-KD HS-5 compared to scrambled siRNA HS-5; which was confirmed by ELISA (Figs. 4b, S14a, S14b). Consistent with this data, ELISA performed on co-culture supernatants from HDAC3-silenced primary BMSCs derived from two patients with MM (one in partial remission, and the other RRMM) showed a 1.6-fold and 1.5-fold increase in sgp130 respectively (Fig. 4b). To establish the source of the increased sgp130, MM1S.Luc cells were first co-cultured with HDAC3 KD or scrambled siRNA HS-5 cells, and then magnetically separated using CD138^+^ beads prior to RNA extraction in both the positive and negative fractions, respectively. RT-PCR showed a 1.3-fold increase in gp130 mRNA in MM1S.Luc cells (CD138 positive fraction) that were co-cultured with HDAC3 KD but not scrambled HS-5 (Fig. S15a). However, gp130 mRNA level in HS-5 cells (CD138 negative fraction) was comparable between the HDAC3 KD and scrambled groups (Fig. S15a). These data suggest that MM cells, and not BMSCs, are the source of increased gp130 secretion in co-culture system. Flow-cytometric analysis showed no changes in surface-bound gp130 (also known as CD130) in MM cells and HS-5 cells after co-culture, regardless of HDAC3 expression, suggesting that CD130 shedding from MM cells may also contribute to increased sgp130 in HDAC3 KD plus MM cell co-cultures (Fig. S15b, 15c).

### Inhibition of the IL6-transignalling pathway is necessary and sufficient to drive HDAC3 KD BMSC-induced MM proliferation arrest

Next, we co-cultured scramble siRNA HS-5 and MM1S.Luc cells in the presence or absence of exogenous human recombinant sgp130 and showed that adding sgp130 to the media was sufficient to block HS-5-induced MM proliferation (Fig. 4c). Vice versa, co-culture of HDAC3 KD HS-5 cells with MM1S.Luc cells in the presence or absence of IL-6/IL-6R protein chimera, the ligand for both cell surface CD130 and decoy receptor sgp130, showed that IL-6/IL-6R was sufficient to rescue HDAC3 KD-mediated MM growth arrest (Fig. 4d). Finally, siRNA KD of CD130 in MM1S.Luc cells caused profound cytotoxicity, suggesting that the IL-6 trans-signaling pathway is necessary for MM survival (Fig. 4e). Altogether, these data suggest that increased level of sgp130, a natural inhibitor of the IL6-trans-signaling pathway may account, at least in part, for HDAC3 KD BMSC-induced MM proliferation arrest. Consistent with inhibition of the IL-6 trans-signaling pathway, western blot analysis of MM1S.Luc cells co-cultured with HDAC3 KD cells showed downregulation of p-STAT3 and p-RB1, with a reciprocal increase of p21 when compared to MM1S.Luc cells co-cultured with scrambled HS-5 BMSC, without changes in ERK and AKT (Fig. S16).

Interestingly, soluble IL-6R was also increased in the supernatant of HDAC3 KD HS-5 co-cultured with MM1S.Luc cells, and exogenous IL-6 was sufficient to rescue HDAC3 KD-mediated MM cell growth inhibition (Figs. 4f, S14a, S14b). Potential mechanisms of this rescue effect include activation of the canonical IL-6 pathway and/or increased IL-6/IL-6R formation. Consistent with published data that increased IL-6 signaling is a mechanism of doxorubicin resistance in MM, HDAC3-silencing in HS-5 cells attenuates cell adhesion mediated drug resistance (CAM-DR) against doxorubicin, but not bortezomib or lenalidomide (Fig. S17a, S17b) [12].

### HDAC3 KD in HS-5 BMSC leads to quantitative changes in exosomes via a paracrine/autocrine loop contributing to MM cell growth arrest

**Fig. 5:**
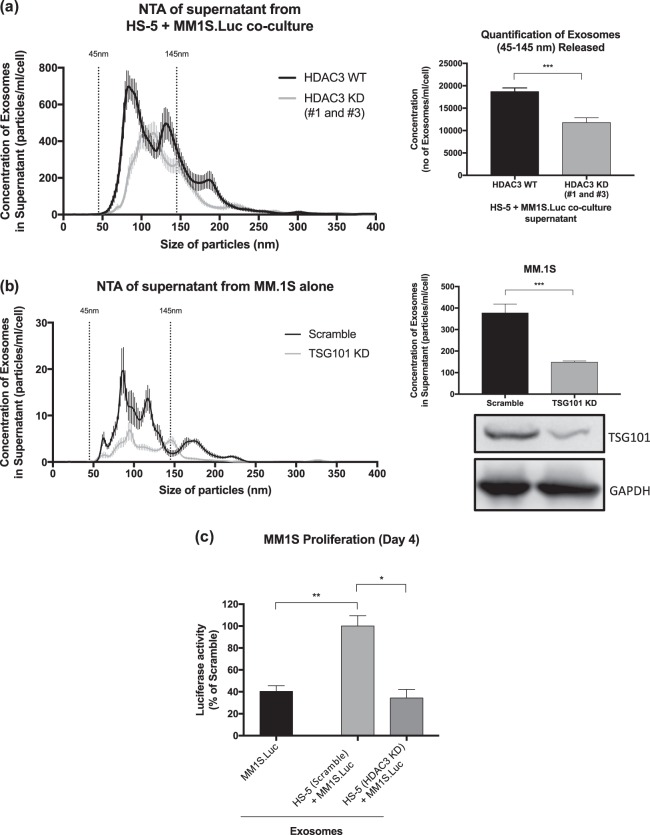
Qualitative and quantitative changes in exosomes derived from HDAC3 KD HS-5 plus MM1S.Luc co-culture supernatant contributes to MM cell growth arrest. **a** Nanoparticle tracking analysis (NTA) showing the mean/modal size and concentration of exosomes in the supernatant of HDAC3 KD HS-5 versus scramble HS-5, co-cultured with MM1S.Luc. The data was normalized to cell count for each condition. **b** siRNA knockdown of TSG101 in MM.1S resulted in decreased exosome secretion as measured by NTA. Data was normalized to cell count for each condition. **c** Exosomes isolated from HDAC3-silenced HS-5 triggers significant MM1S.Luc growth inhibition compared to exosomes isolated from scramble siRNA HS-5 (65.7% decrease in MM1S.Luc proliferation in HS-5 (HDAC3 KD) alone, *P* < 0.05)

As exosomes have been shown to play a role in MM pathogenesis, we next asked whether they play a role in mediating HDAC3 KD BMSC-induced MM proliferation arrest. To this end, we performed exosome enrichment from conditioned media obtained from co-culture of MM1S.Luc cells with HDAC3 KD versus scramble siRNA HS-5 cells. Quality of enrichment for exosomes was confirmed using western blot and nanoparticle tracking analysis (NTA) (Fig. 5a, S18a). We observed a 20% decrease in the quantity of exosomes secreted in HDAC3 KD versus scrambled siRNA HS-5 co-culture supernatants, suggesting that HDAC3 KD in HS-5 cells leads to a reduction in exosome secretion in co-culture systems (Fig. 5a). When comparing NTA profiles, we discovered that HDAC3 KD in HS-5 BMSC markedly decreased small exosome vesicles (Exo-S, 60–80 nm), while sparing large exosome vesicles (Exo-L, ~120 nm) (Fig. 5a). Consistent with the MM-BMSC paracrine-autocrine signaling hypothesis, HDAC3 KD in HS-5 BMSC alone did not show any quantitative change in exosome secretion when compared to scramble siRNA HS-5 BMSC, further supporting the paracrine-autocrine nature of this crosstalk (Fig. S18b–d).

To define the molecular mechanisms underlying the quantitative changes observed in the exosome pool, we performed proteomic profiling of cell lysates from MM1S.Luc cells co-cultured with either HDAC3-silenced or scramble siRNA HS-5 cells (Table S1; Fig. S19a–c). Interestingly, the expression of tumor susceptibility gene 101 (TSG101), a protein relevant in exosome biogenesis, is induced in MM1S.Luc by co-coculture with scramble siRNA HS-5 cells (Fig. S18e). However, HDAC3 KD in HS-5 prior to co-culture completely abrogates BMSC-induced TSG101 induction in MM1S.Luc (Table S1; Fig. S18e). To assess whether downregulation of TSG101 is sufficient to reduce exosome secretion, we performed TSG101 KD in the MM cell lines MM.1S and RPMI-8226 (Figs. 5b, S18f). Our data show that TSG101 KD in both MM.1S and RPMI-8226 leads to reduced Exo-S secretion. Altogether, these data suggest that HDAC3-KD in HS-5 BMSC inhibits BMSC-induced upregulation of TSG101 in MM cells, resulting in decreased exosome release.

### HDAC3 KD in HS-5 BMSC leads to qualitative changes in exosomes via a paracrine/autocrine loop contributing to MM cell growth arrest

In order to assess qualitative changes in exosomes, we next cultured MM cells in the presence of 50ug/ml exosomes harvested and enriched from either HDAC3 KD or scrambled siRNA HS5-MM co-cultures. First, we confirmed exosome internalization in MM cells via confocal microscopy and flow-cytometry (Fig. S20a, S20b). Next, we observed 2.5-fold increased proliferation in MM cells cultured in the presence of exosomes derived from scramble HS5-MM co-cultures, compared to MM cells alone (Fig. 5c). However, this proliferative burst was abrogated by 66% when MM cells were cultured with exosomes derived from HDAC3 KD HS5-MM co-cultures (*P* value < 0.05, Fig. 5c). These data indicate that HDAC3-silencing in HS-5 BMSCs leads to qualitative changes in exosomes derived from BMSC-MM co-cultures, which result in decreased MM proliferation.

### Exosomes derived from HDAC3 KD BMSC show downregulation of pro-survival microRNA (miR) miR380, miR382, miR15b, miR9986, and miR5191

We next performed small RNASeq on exosomes isolated from the conditioned supernatant of MM1S.Luc cells alone, HDAC3 KD HS-5-MM1S.Luc co-culture, or scrambled siRNA HS-5-MM1SLuc co-culture. Compared to MM cells alone, exosomes from MM1S.Luc cells co-cultured with scrambled HDAC3 HS-5 showed increased expression of a set of miRs which are known to exert a pro-survival function; including miR380, miR382, miR15b, miR9986, and miR5191. Importantly, upregulation of these miRs was partially abrogated in HDAC3 KD HS-5-MM co-culture (Fig. S21a, S21b). Taken together, these data indicate that HDAC3 KD in HS-5 cells leads to downregulation of pro-survival miRNAs in exosomes obtained from BMSC-MM co-culture systems.

### HDAC3 KO in HS-5 abrogates BMSC-induced proliferation of MM cells in a 3D-in vitro co-culture model and significantly impairs tumor growth in vivo

**Fig. 6:**
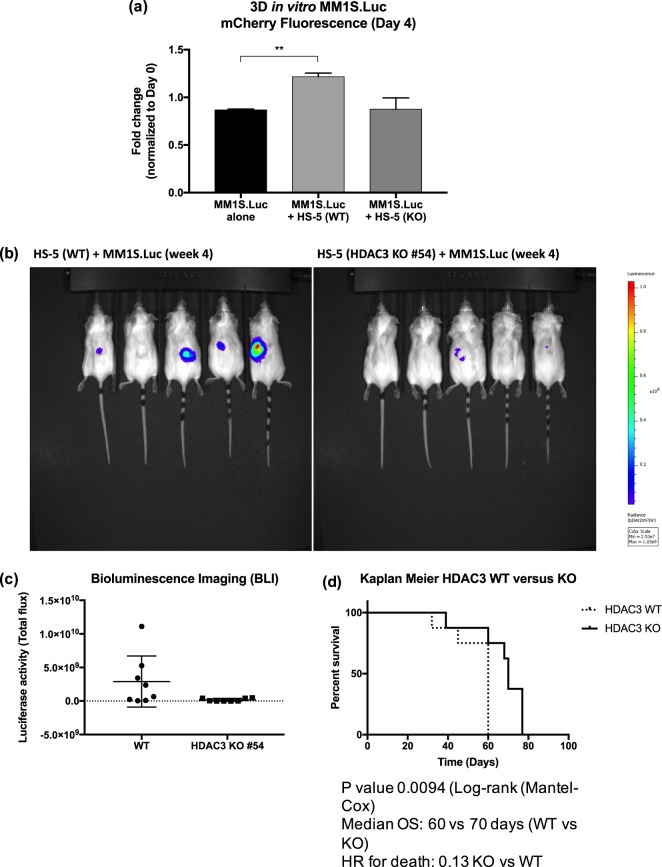
HDAC3 expression in BMSC is essential for its MM growth-supporting effects both in a *3D*-in vitro co-culture model and in vivo. **a** MM1S.Luc proliferation in a 3D system alone or in co-culture with WT or HDAC3 KO HS-5. **b** Bioluminescence imaging of mice inoculated subcutaneously with MM1S.Luc cells and WT (left panels) or HDAC3 KO (right panels) HS-5. Five representative mice per group are shown. **c** Luciferase activity of tumor in each cohort was quantified using the Living Image software. Data represent mean ± s.d. (*P* value > 0.05). **d** The overall survival of mice inoculated with HDAC3 KO HS-5 plus MM1S.Luc was significantly higher compared to mice inoculated with WT HS-5 plus MM1S.Luc (median OS: 70 (WT) vs. 60 (KO) days; *P* = 0.0094) (*N* = 8 each group)

To mimic the MM BM niche, MM1S.Luc cells were co-cultured with HDAC3 WT or KO HS-5 cells in a hydrogel 3D in vitro co-culture system. As expected, WT HS-5 cells induced a 1.4-fold increase in MM1S.Luc proliferation when compared to MM cells alone. However, this effect was completely abrogated when MM1S.Luc cells were co-cultured with HDAC3 KO HS-5 cells (*P* value < 0.05, Fig. 6a). These data suggest that targeting HDAC3 in BMSC leads to significant abrogation of BMSC-induced MM proliferation in a 3D system.

To evaluate the translational significance of our findings, we inoculated immunodeficient mice subcutaneously with a 1:1:1 mix of MM1S.Luc cells, Matrigel and either HDAC3 WT or KO HS-5 cells. At four weeks post inoculation, mice bearing HDAC3 KO tumor showed impaired engraftment (50% vs. 25% non-engrafted tumor in HDAC3 KO and HDAC3 WT group, respectively) and decreased tumor burden (mean total flux 1.1e7 vs. 2.9e9 in HDAC3 KO and WT, respectively), (Figs. 6b, c). Longitudinal follow-up of mice revealed that HDAC3 KO HS-5-tumor bearing animals had statistically significant longer survival compared to HDAC3 WT HS-5 tumor bearing animals (median OS 60 vs. 70 days; HDAC3 WT vs. HDAC3 KO respectively, *P* value = 0.009; hazard ratio for death 0.13 KO vs. WT) (Fig. 6d). These data support a role for HDAC3 targeting in BMSC as a novel strategy to impact MM survival and proliferation in vivo.

## Discussion

Unlike solid tumors where primary and metastatic disease occur in separate and distinct sites, MM is characterized by the widespread involvement of multiple sites within the BM microenvironment [13]. It is well established that, through bi-directional signaling loops, the BM microenvironment induces MM cell proliferation, survival, drug resistance, and dissemination [13–15]. Indeed, agents most effective in the treatment of MM not only target cancer cells but also the BM niche, including immunomodulatory drugs and proteasome inhibitors [8, 16, 17]. Identifying novel agents exerting anti-MM activity in the context of the BM niche is therefore crucial in developing effective therapies. We have previously shown that the HDAC3-selective inhibitor BG45 inhibits MM proliferation [6, 18]. To date, however, the biological impact of HDAC3 inhibition in the context of the BM microenvironment has not yet been fully elucidated. We here therefore examined whether HDAC3 inhibition in the BMSC compartment could exert an indirect effect on MM cells.

Neo-angiogenesis is a major determinant of MM progression, dissemination, and drug resistance. Previous reports have shown that HDAC inhibitors inhibit angiogenesis by attenuating endothelial tube formation; however, the specific HDAC isoform(s) that mediate this effect have not been defined [11, 19, 20]. Importantly, our study shows that HDAC3 KD in BMEC abolishes endothelial tube formation, suggesting that this isoform is relevant in supporting neo-angiogenesis in the MM BM niche.

**Fig. 7:**
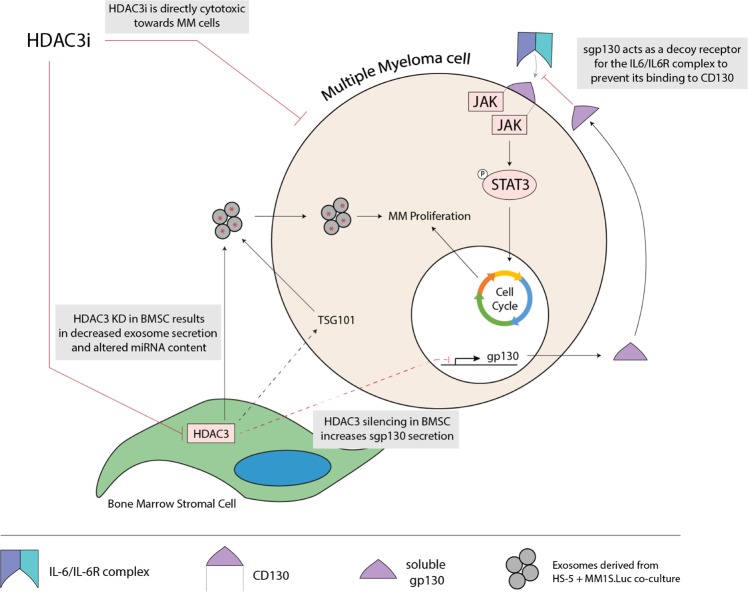
Schema of proposed molecular mechanisms underlying anti-MM effect of HDAC3 targeting in BMSC. On one hand, HDAC3 targeting in BMSC has anti-MM effect by increasing sgp130 secretion, via a paracrine-autocrine loop, which acts as a decoy receptor to prevent binding of the IL6/IL6R complex to CD130. The resulting abrogation of IL-6 trans-signaling and downstream STAT3 signaling leads to decreased MM proliferation. On the other, HDAC3 silencing in BMSCs leads to decreased exosome secretion associated to downregulation of TSG101 and qualitative changes in miRNA content, resulting in MM growth inhibition

We then focused our attention to BMSCs. First, we observed that HDAC3 expression is increased in MM patient-derived BMSC compared to healthy donor-derived BMSC, and that HDAC3 expression in BMSC can be induced by MM co-culture in vitro. By using BMSC lines and patient cells, including autologous BMSC and MM cells, we show that targeting HDAC3 via KD, KO, or pharmacological inhibition results in decreased MM cell proliferation. Focusing on the secretome of MM-BMSC co-culture systems, we identified increased sgp130 and consequent inhibition of IL6 trans-signaling pathway as a major molecular mechanism driving HDAC3 KD HS-induced MM proliferation arrest. Previous studies have reported sgp130 to be the natural inhibitor of the IL-6 trans-signaling pathway, a pathway that we and others have shown to mediate MM proliferation [9, 21]. Specifically, the IL-6 trans-signaling pathway involves the binding of IL-6 with soluble IL6R to form the IL6/IL-6R complex. The complex can then binds to CD130 on MM cells to activate downstream STAT3 signaling which in turns mediates MM cell survival and proliferation [21]. Conversely, sgp130 acts as a decoy receptor to prevent binding of the IL-6/IL-6R complex to membrane CD130, thereby inhibiting downstream STAT3 signaling and MM cell proliferation (Fig. 7) [21]. Consistent with this mechanism, our results demonstrate that: CD130 knockdown was profoundly cytotoxic to MM cells; excess sgp130 is sufficient to recapitulate HDAC3 KD HS-5 induced growth arrest; and inhibition of IL6 trans-signaling is necessary to mediate HDAC3 KD HS-5 induced growth arrest.

Together with cytokines, exosomes are the main soluble mediators of intercellular communication. Previous reports showed that exosomes derived from both BMSCs and MM are able to promote MM growth [22–24]. In this study, we show that exosome secretion is reduced in HDAC3 KD HS5-MM co-cultures due to downregulation of TSG101 in MM. TSG101 is an integral component of endosomal sorting complex required for transport (ESCRT) complex, which drives exosome biogenesis and is a recognized exosome marker [25]. We and others have previously reported that silencing of TSG101 results in reduced exosome secretion [25, 26]. Importantly, recent studies have revealed that exosomes are able to mediate the intercellular exchange of miRNAs in the context of the MM BM microenvironment [27]. In our study, exosome miRseq revealed a decrease in the exosomal expression of miR380 and miR382 when HDAC3 was silenced in BMSCs. miR380 has been reported to attenuate p53 signaling, while miR382 has been reported to be more highly expressed in patients with MM, suggesting a pathogenetic function of these miRNAs [28, 29].

To further validate the potential clinical significance of our findings, we next used a 3D in vitro system to show that HDAC3 KO in BMSCs significantly impacts MM cell proliferation in the co-culture setting. Finally, we show that HDAC3 KO in BMSC impairs tumor engraftment and growth in a murine xenograft model of human MM, further supporting the potential clinical utility of HDAC3 inhibition to target MM cells in the context of the BM niche.

In summary, we have previously shown that targeting HDAC3 directly impacts MM cell survival, and here provide a phenotypic and molecular characterization of the effects of targeting HDAC3 in the MM BM niche. By using in vitro and in vivo models, we show that disrupting HDAC3 signaling in BM accessory cells negatively affects MM growth and survival in the MM BM microenvironment. This is the first report of the isolated effects of HDAC3 targeting in BM accessory cells, showing such an indirect antitumor effect. Most importantly, our study further supports the clinical development of drugs targeting not only tumor cells, but also the microenvironment, in an effort to limit drug resistance and increase therapeutic efficacy. Our results therefore provide the preclinical rationale for clinical trials targeting HDAC3 in BMSCs to indirectly inhibit tumor cell growth, survival, and drug resistance in the BM milieu and thereby improve patient outcome.

## Supplementary information

Supplementary Data

Supplementary Table 1
