# Supplementary material for: Targeting histone deacetylase 3 (HDAC3) in the bone marrow microenvironment inhibits multiple myeloma proliferation by modulating exosomes and IL-6 trans-signaling
Source: Leukemia. 2019 May 29;34(1):196–209. doi: 10.1038/s41375-019-0493-x (PMC6883144; doi:10.1038/s41375-019-0493-x)
Supplement: Supplementary file 1 — Supplementary Data [file 41375_2019_493_MOESM1_ESM.pdf]

SUPPLEMENTARY METHODS

Cell culture

MM.1S and H929 MM cell lines expressing luciferase and RFP (MM1S.Luc and H929.Luc) were kindly gifted by Dr Xue Li (Harbin Institute of Technology, Harbin, China). The bone marrow stromal cell line (BMSC) HS-5 was purchased from the American Type Culture Collection (ATCC). The MM-derived stromal cell line MSP-1 was kindly gifted by Dr Abdel Kareem Azab (Washington University in Saint Louis School of Medicine, USA). HS-5 and MSP-1 were maintained in Dulbecco's Modified Eagle's Medium (DMEM) containing 100 U ml^{-1} penicillin and 100 ug/ml streptomycin, supplemented with 10% (v/v) fetal bovine serum. The human bone marrow endothelial cell line, BMEC-60, was kindly provided by Dr CE van der Schoot (CLB, Amsterdam, The Netherlands), and was cultured in endothelial culture medium (EGM-2, Lonza) with full supplements (EGM-2 bullet kit: 2% FBS, 0.4% hFGF-2, 0.1% VEGF, 0.1% R^3-IGF-1, 0.1% hEGF, 0.04% hydrocortisone, 0.1% ascorbic acid, 0.1% heparin, and 0.1%-GA-100). All MM cell lines were maintained in Roswell Park Memorial Institute (RPMI)-1640 medium containing 2.5 mg ml^{-1} plasmocin, 100 U ml^{-1} penicillin and 100 ug ml^{-1} streptomycin, supplemented with 10% (v/v) fetal bovine serum and 2 µg L-glutamine, in 5% CO_2 at 37ºC. Cell lines were intermittently tested to rule out mycoplasma contamination using the MycoAlert Mycoplasma Detection Kit (Lonza), and MM cell lines were authenticated via short tandem repeat (STR) profiling.

Primary bone marrow stromal cells (BMSCs)

Bone marrow samples were obtained from patients diagnosed with MM, after obtaining informed consent and approval by the Institutional Review Board of the Dana-Farber Cancer

Institute. Bone marrow mononuclear cells (BMMC) were separated by Ficoll-Paque PLUS (GE Healthcare), and MM cells were enriched by CD138-positive selection with anti-CD138 magnetic activated cell separation microbeads (Miltenyi Biotec). Long-term bone marrow stromal cell culture was established by culturing CD138-negative bone marrow mononuclear cells for 4-6 weeks in RPMI containing 100 U ml^{-1} penicillin and 100 ug ml^{-1} streptomycin, supplemented with 20% (v/v) fetal bovine serum (FBS).

Autologous co-culture of primary $CD138^+$ MM cells and HDAC3-silenced $CD138^-$ BMMCs derived from patients with MM

BMMCs were first isolated from bone marrow aspirates using Ficoll-Paque PLUS (GE Healthcare). MM cells were then magnetically separated through CD138-positive selection. The $CD138^-$ fraction was then stained with CellTrace™ Violet (Thermo Fisher) according to the manufacturer's protocol. Following staining, the NEON transfection system (Thermo Fisher) was used to transfect $CD138^-$ BMMCs with HDAC3 siRNA. Briefly, 75nM of siRNA was used for each transfection. The following electroporation parameters were used. Pulse voltage: 2150V, pulse width: 20ms, pulse number: 1, cell density 2 x 10^7 cells ml^{-1}. CD138+ and transfected CD138- BMMM were cultured separately overnight. The following day, CD138- BMMC were washed once with PBS and co-cultured with autologous $CD138^+$ MM cells for up to 7 days. Cells were then collected and stained with Annexin V and PI before analysis by flow cytometry. CD138+ (cell-trace violet negative) and CD138- (cell-trace violet positive) cells were subsequently discriminated by gating according to expression of cell trace violet (Y axes).

Lentiviral transduction of HDAC3 shRNA

Viral production and infection were carried out as previously described [9]. In competent lentivirus was produced by transiently transfecting host 293T cells with pLKO.1 plasmid expressing HDAC3 shRNA or scrambled sequence, psPAX2, and pMD2.G in a 4:1:1 ratio using Lipofectamine 2000 Transfection Reagent (Invitrogen, CA, USA), according to the manufacturer's instructions. MM cells were incubated in lentivirus containing media in the presence of 8μg/ml of polybrene (Sigma-Aldrich) for 48H. After 48H of viral infection, cells expressing shRNAs were selected with puromycin dihydrochloride (Sigma-Aldrich) at 0.5μg/mL. The following shRNAs (Dharmacon™, GE Healthcare Life Sciences) against CD130 were used: #1 TRCN0000058283 (GCCACATAATTTATCAGTGAT), #2 TRCN0000058284 (CCAGTCCAGATATTTCACATT), #3 TRCN0000058285 (CCCATACTCAAGGCTACAGAA).

Endothelial tube formation assay (In-vitro angiogenesis assay kit; Abcam)

The bone marrow endothelial cell line (BMEC60) was transfected with HDAC3 siRNA for 48H to silence HDAC3. Cells were then harvested, washed, and re-suspended in endothelial cell media (Lonza). 50ul of thawed extracellular matrix solution (EMS) was then added to each well of a pre-chilled (on ice) 96-well plate sterile culture plate. The plate was incubated for 1H at 37ºC to allow the solution to form a gel. 2×10^4 cells/well was added onto the solidified extracellular matrix gel and incubated overnight. The incubation media was then carefully removed by aspiration, without disturbing the cells or the extracellular matrix gel. The wells were then gently washed with 100ul of wash buffer to remove serum. Staining dye was added, and cells were incubated

for 30mins at 37ºC. Endothelial tube formation was examined using light and fluorescence microscopy.

Flow cytometry apoptosis assay

Cells were harvested following RNAi/drug treatment and washed twice with PBS before being pelleted by centrifugation at 1200 rpm. The cell pellet was re-suspended in PBS containing 1X Annexin V binding buffer with 1 mg/ml of Annexin V antibody (BD Biosciences). Cells were stained with Annexin V for 20 mins on ice. Prior to acquisition, 1mg/ml of propidium iodide (or DAPI) was added, and cells were acquired using a flow cytometer (BD LSRFortessa™) and analyzed using FlowJo (TreeStar). Cells that were propidium iodide (PI) negative and Annexin V negative are considered alive; PI negative and Annexin V positive cells were considered early apoptotic; double positive cells were considered late apoptotic; and PI positive but annexin V negative cells were considered necrotic.

MM cell adhesion assay

Bone marrow stromal cells (BMSCs) were plated at a density of 1 x 10^4 per well in a 96-well plate 1 day before the adhesion assay. Human fibronectin- or BSA-coated plates were purchased from R&D Systems. MM cells were pre-labeled by incubating in the presence of 1 mM calcein AM (Molecular Probes) at 37 ºC for 30 min. After washing with PBS with calcium and magnesium (PBS (+)) twice, labeled cells were resuspended in PBS (+) at a concentration of 1 x 10^6 ml^{-1}. Subsequently, 1 x 10^5 cells were then incubated in 96-well plates coated with bone marrow stromal cells (BMSCs), fibronectin or BSA at 37ºC for 2H. After measuring the fluorescent intensity

of pre-wash samples, wells were washed two to three times with PBS. The fluorescent intensity of adherent cells was then quantified using a fluorescence plate reader (SpectraMax M3, Molecular Devices) at an excitation wavelength of 485 nm and an emission wavelength of 525 nm. The percentage adhesion was determined by calculating the ratios of the fluorescent intensity of the post-wash sample to that of the pre-wash sample.

Transwell migration assay

For migration studies, control or HDAC3 silenced HS-5 cells were plated on the lower chambers and cultured in serum-free RPMI 1640 overnight. The next day, control or 15ug/ml of anti-CXCL1 neutralizing antibody (R&D systems) was added to the lower chamber before MM1S.Luc (5 x 10^5 cells) suspended in serum-free RPMI 1640 media were placed in the upper chambers of the transwell plates (pore size 8µM; Costar-Corning). After 4H incubation at 37ºC, MM1S.Luc cells that had migrated to the lower chambers were lysed, and the quantitative Luciferase assay was performed.

Protein array

Analysis of the expression of cytokines in co-cultures of multiple myeloma cells and bone marrow stromal cells was carried out using the RayBio C1000 antibody array (Redwood City, USA). The membrane, which was spotted with various immobilized antibodies for cytokines and growth factors, was incubated in the presence of 2ml of conditioned media from co-cultures of MM1S.Luc and HS-5 cells. The membrane was then incubated with a mixture of biotin-conjugated anti-cytokine antibodies (dilution, 1:60). Antibodies bound to the array were detected using

streptavidin-horseradish peroxidase according to the manufacturer's instructions, and then developed with enhanced chemiluminescence.

sgp130 assay

ELISA detection of soluble gp130 was carried out using a murine anti-human gp130 antibody for capture and a polyclonal antibody against human gp130 conjugated with horseradish peroxidase for detection, according to the manufacturer's instructions (R&D Systems).

Quantitative proteomic profiling by label-free liquid chromatography–MS/MS analysis

Cell pellets were sonicated in 8 M urea/50 mM NH4HCO3/0.1% ProteaseMax using sonication, with resultant protein levels quantified using a Bradford assay. Following dithiothreitol (DTT) reduction and iodoacetic acid-mediated alkylation, a double digestion was performed using Lys-C (for 4H at 37 °C) and Trypsin (overnight at 37 °C) on 5 µg of protein. The samples were then desalted prior to analysis using C18 spin columns (Thermo Scientific), and 500ng was loaded onto an Ultimate 3000 NanoLC system (Dionex Corporation, Sunnyvale, CA, USA) coupled to a Q-Exactive mass spectrometer (Thermo Fisher Scientific). The raw data were analyzed using Progenesis QI for Proteomics software (version 3.1; Non-Linear Dynamics, a Waters Company, Newcastle upon Tyne, UK), as previously described, with following modifications: the filter mass peaks with charge states from +1 to+6 was applied to the MS/MS data files; peptides were identified using taxonomy: Homo sapiens in the SwissProt database, and peptides with XCorr scores 41.9 for +1 ions, 42.2 for +2 ions, and 43.75 for +3 ions or more (from Sequest HT) were selected. Three biological replicates were performed.

Bioinformatic analysis

For interpretation of MS/MS spectra, we performed STRING protein-protein interaction (string-db.org) with enrichment of Gene Ontology (GO) analysis of these data sets to predict biological processes, as well as interactive networks to identify signaling pathways for identified genes.

Exosome isolation

Cells were cultured for 48H in RPMI-1640 with 10% exosome-depleted FBS (Thermo Fisher). The supernatant was then harvested by centrifugation at 2,000 rpm, and filtered through a 0.2uM filter to remove cell debris. The exoEasy Maxi kit from Qiagen was then used to isolate exosomes, according to the manufacturer's protocol. A buffer exchange using size exclusion chromatography was performed to remove toxic buffer XE prior to co-culture with MM1S.Luc cells.

Exosome RNA isolation

Exosomes were first isolated from conditioned supernatant using the exoEasy Maxi kit (Qiagen). Instead of eluting the exosomes with buffer XE, 700ul of Qiazol was added to the membrane. 3.5ul of miRNeasy Serum/Plasma Spike-In Control was added to each sample. 90ul of chloroform was added to the lysate and shaken vigorously for 15s. Following centrifugation, the upper aqueous phase was transferred to a new collection tube, and 2 volumes of 100% ethanol was added. Total RNA from the resultant sample was then isolated using the RNeasy MinElute spin column.

Nanoparticle tracking analysis (NTA)

EV samples were run using the standard measurement procedure at 25 °C with a constant syringe infusion rate of 50, per the NanoSight NTA 3.1 Software (Malvern). The data for each sample were obtained from 10 independent 60s video captures on the NS300, analyzed, and normalized to final cell counts for each condition.

Small RNAseq

Small RNAseq of total RNA from exosomes was performed using the Illumina HiSeq, 2x150bp configuration (Genewiz Next Generation Sequencing Services, South Plainfield, NJ). RNAs <200bp were then selected for further analysis by the Joslin Diabetes Center Bioinformatics core (Boston, MA).

Confocal microscopy

Exosome samples were stained according to the following protocol: 15ul of EV-containing PBS solution was pipetted onto 15ul of a 40uM CFSE solution, and incubated for 2H at 37ºC. Tubes were mixed by flicking every hour. The 8 samples were then combined, and unbound CFSE was removed by size exclusion chromatography. CFSE-labeled exosomes were then cultured for 6h with MM1S.Luc-mCherry at 37ºC and were washed twice with PBS. After washing, cells were cytocentrifuged onto glass slides at 800 rpm for 5min and allowed to air dry. Cells were then fixed with 1% paraformaldehyde at room temperature for 30min. After washing in PBS, cells were then permeabilized with 0.1% Triton X-100 for 3min, and washed with PBS. DNA was stained with 1ug/ml of 4',6-diamidino-2-phenylindole (DAPI) for 5min and washed in PBS. Coverslips were

mounted using DakoCytomation fluorescent mounting medium. Stained cells were imaged using a Zeiss confocal microscope, and images were captured using the AxioVision Imaging software.

Synthesis of methacrylated hyaluronic acid (HAMA)

2-hydroxy-1-[4-(hydroxyethoxy)phenyl]-2-methyl-1-propanone (Irgacure 2959) was obtained from BASF Corporation (Florham Park, NJ). Microscope slides were supplied by VWR (Radnor, PA). Methacrylic anhydride and sodium hydroxide were obtained from Sigma-Aldrich (St. Louis, MO). Sodium hyaluronate was supplied by Lifecore Biomedical (Chaska, MN). All reagents were used as received without further purification. To synthetize methacrylated hyaluronic acid (HAMA), 1 g sodium hyaluronate was added to 100 mL of deionized water and completely dissolved. To this solution, 1 mL methacrylic anhydride was added at 4 °C under moderate stirring conditions at 150 rpm. The reaction was carried out for 24 h. The pH of the reaction mixture was maintained between 8 and 10 using 5 M sodium hydroxide throughout the reaction period. The resulting solution was dialyzed against deionized water for 3 days in 12-14 kDa molecular weight cut-off membrane at 4 °C. The solution was then frozen at -80°C, lyophilized using a freeze dryer, and stored at -80 °C until experimental use. The degree of methacrylation for HAMA was determined to be 16% through proton nuclear magnetic resonance (NMR) analysis.

Cell encapsulation in methacrylated hyaluronic acid (HAMA) hydrogels in three dimensions (3D)

The prepolymer solution for three-dimensional (3D) cell encapsulation was prepared by dissolving 2% (w/v) HAMA and 0.5% (w/v) photoinitiator (Irgacure 2959) in DPBS at 70 ˚C. The

prepolymer solution was then kept in 37˚C incubator until the cell encapsulation process. The MM1S cells were cultured in RPMI 1640 medium supplemented with 10% (v/v) FBS, 1X GlutaMAX, and 1% (v/v) penicillin/streptomycin. The HS-5 cells were cultured in low glucose DMEM medium supplemented with 10% (v/v) FBS, 1X GlutaMAX, and 1% (v/v) penicillin/streptomycin. These cells were cultured in a humidified incubator at 37˚C with 5% CO^2 supplementation. The HS-5 wild type (WT) and HS-5 knockout (KO) cells were trypsinized and centrifuged at 1500 rpm to obtain cell pellet. The MM1S.Luc cells were collected using a cell scraper and then centrifuged. Three different conditions for 3D cell encapsulation was performed: MM1S.Luc only; MM1S:HS-5 (WT) co-culture at 1:1 cell ratio; and MM1S:HS-5 (KO) co-culture at 1:1 cell ratio. All cells were counted and resuspended in the prepolymer solution at 5 million cells per mL cell density for all three conditions. Ten microliters of the prepolymer solution with cells was next placed on a petri dish that included 150 µm spacer, and covered with a glass slide. The sample was exposed to UV light at 2.5 mW/cm^2 power for 4s to photo-crosslink the cell-laden prepolymer solution. The resulting hydrogel was then removed from the petri dish, rinsed in DPBS to remove the unreacted polymer, and subsequently cultured in a 24-well plate for up to 4 days. Three to four replicates were performed for each condition.

Measuring 3D *in vitro* MM1S.Luc proliferation using mCherry fluorescence

3D cell encapsulation in 2% methacrylated hyaluronic acid (2% HAMA) was performed for three different conditions: MM1S.Luc alone, MM1S.Luc:HS-5 (WT) co-culture, and MM1S.Luc:HS-5 (KO) co-culture. The resultant hydrogel was then subsequently cultured in a 24-well plates for up to 4 days. MM1S.Luc proliferation was then measured at day 0 and day 4 by reading mCherry

fluorescence (Ex: 587/Em: 610) using a spectrophotometer. Readings at day 4 were normalized to readings at day 0. MM1S.Luc proliferation was assessed by reading mCherry fluorescence (Ex: 587/Em: 610) using a spectrophotometer on days 0 and 4, according to the manufacturer's protocol. Four replicates were used for each condition.

Immunoblotting

Cells were harvested and lysed using sodium dodecyl sulfate-polyacrylamide gel electrophoresis (SDS-PAGE) sample buffer containing 60 mM Tris-HCl, pH 6.8, 2% SDS, 10% glycerol, 0.005% bromophenol blue, 5 mM ethylenediaminetetraacetic acid, 5 mM NaF, 2 mM Na_3VO_4, 1 mM phenylmethylsulfonyl fluoride (PMSF), 5 μg/mL leupeptin, and 5 μg/mL aprotinin; and then heated at 100 °C for 5 min. After the determination of protein concentration using DC protein assay (Bio-Rad, Hercules, CA), β-mercaptoethanol (β-ME) was added to the whole-cell lysates to a 2% final β-ME concentration. The whole-cell lysates were subjected to SDS-PAGE, transferred to nitrocellulose membranes (Bio-Rad, Hercules, CA) or polyvinylidene fluoride membranes (Millipore, Billerica, MA), and immunoblotted -HDAC1 (#34589S), -HDAC2 (#57156S), -HDAC3 (#3949S), -Acetyl-histone H3 (lysine 9) (Ac-H3K9) (#9649S), -glyceraldehyde-3-phosphate dehydrogenase (GAPDH) (#2118S), -Signal transducers and activators of transcription 3 (STAT3) (#30835S), -phospho-STAT3 (pSTAT3) (tyrosine 705) (#9131S), -Akt (#9272S), -phospho-Akt (serine 473) (#9271S), -p21 (#2947S), -Rb (#9309S), -phospho-Rb (serine 807/811) (#8516S), -GP130 (#3732S) (Abs; Cell Signaling Technology, Beverly, MA).

SUPPLEMENTARY TABLE LEGEND

Table S1: Results of mass spectrometric analysis of proteins in HDAC3-silenced vs control HS-5 cultured alone or together with MM cells

Mass spectrometric analysis was performed on cell lysates obtained from HDAC3-silenced vs control HS-5 cultured alone or together with MM cells. In the HS-5 and MM co-culture group, CD138 positive selection was performed to separate the HS-5 cells from the MM cells before each compartment was sent for proteomic sequencing.

SUPPLEMENTARY FIGURE AND FIGURE LEGENDS

Figure S1

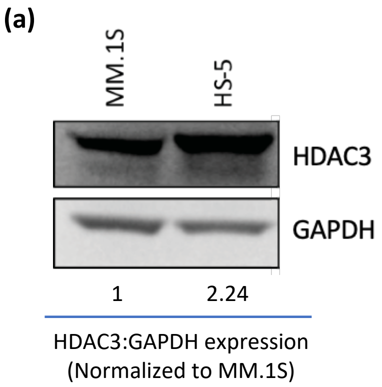

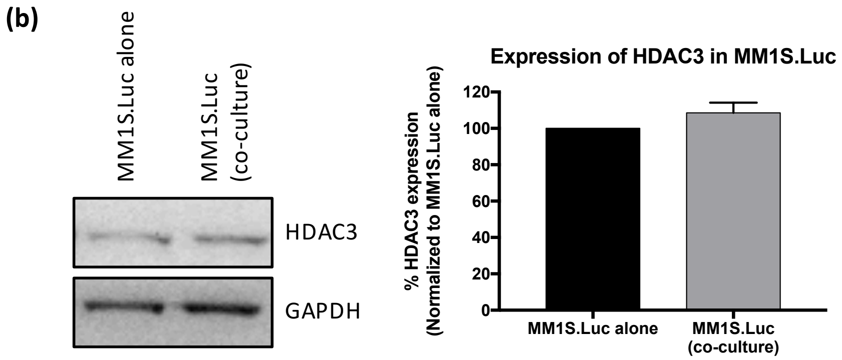

(c) **HDAC3 TPM in MM**
(alone or co-culture with HS-5)

INA6	INA6+HS5	MM1S	MM1S+HS5	RPMI	RPMI+HS5
12.95	11.18	15.91	13.28	8.62	7.62

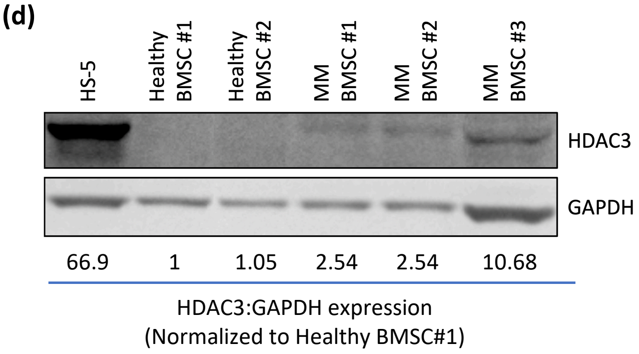

Figure S1: HDAC3 expression in MM cells is not upregulated by co-culture with BMSCs

(a) Western blot showing relative expression of HDAC3 in MM.1S MM cell line vs HS-5 stromal cell line. Notably, HDAC3 is more highly expressed in HS-5 compared to MM.1S as quantified using ImageJ. **(b)** HDAC3 expression in MM1S.Luc is not significantly altered when MM1S.Luc is co-cultured with HS-5. **(c)** Gene expression analysis from the IFM/DFCI dataset showing the relative expression of HDAC3 a panel of MM cell lines cultured alone or together with HS-5. **(d)** Western blot showing increased HDAC3 expression in primary MM-BMSC (N=3) versus HD-BMSC (N=2). Protein lysate from HS-5 was used as positive control. GAPDH served as loading control. Quantification was performed using ImageJ.

Figure S2

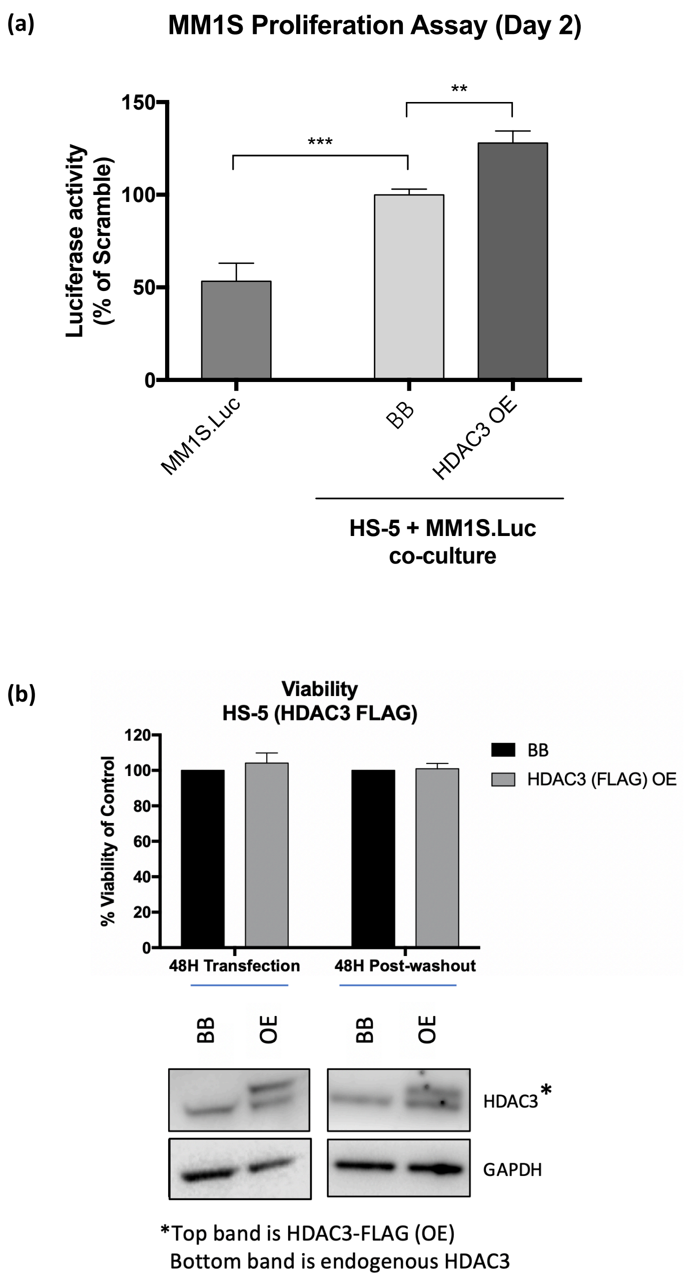

Figure S2: HDAC3 overexpression in HS-5 significantly increased MM cell growth in co-culture

(a) and (b) HDAC3 overexpression in HS-5 significantly increased MM cell growth in co-culture (27.9% increase in MM proliferation in HDAC3 OE, $p < 0.05$) without affecting HS-5 viability. **(b)** Western blot showing successful overexpression of HDAC3-FLAG at indicated times.

Figure S3

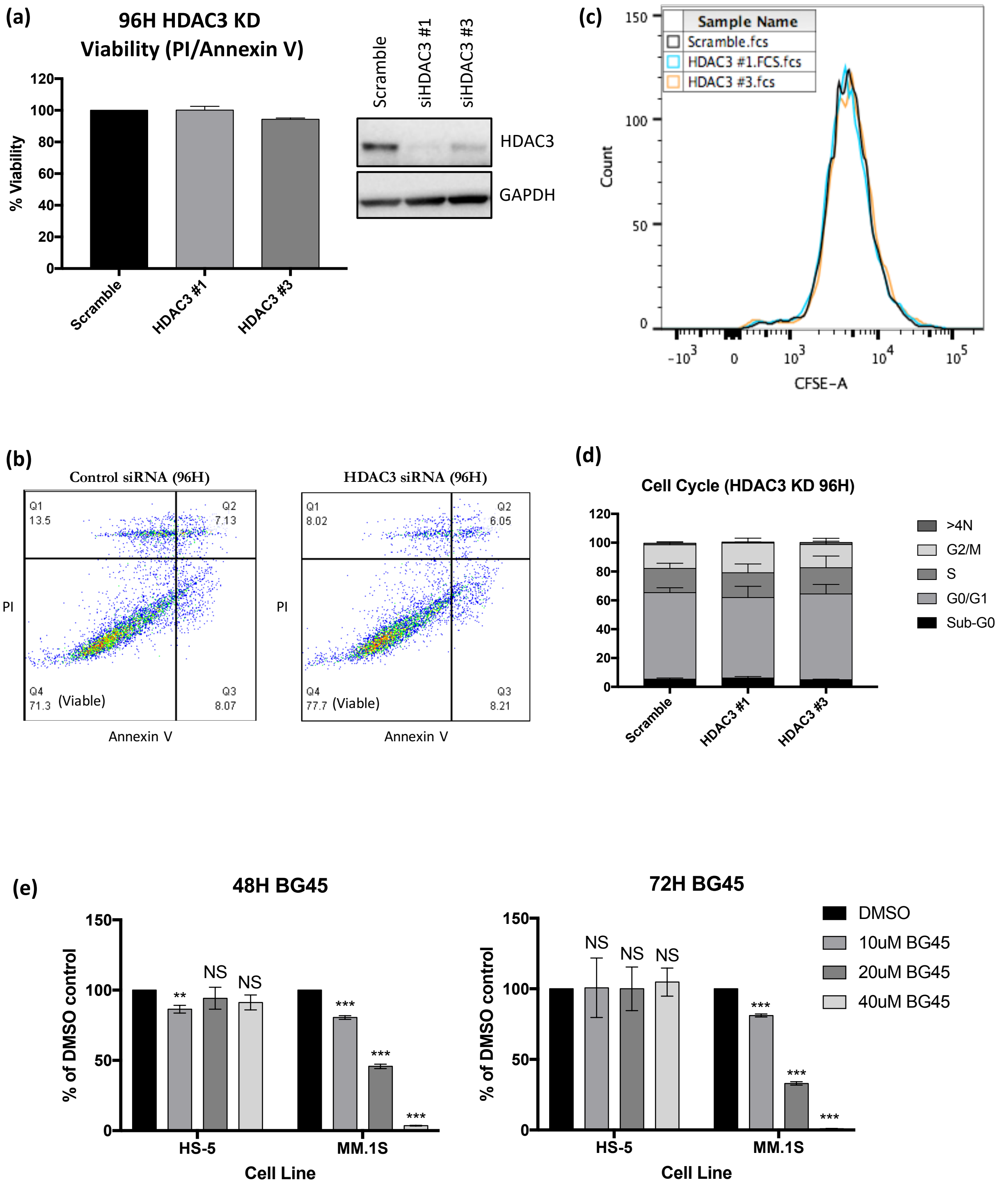

263

Figure S3: HDAC3 expression is not necessary for BMSC viability or proliferation.

(a) HDAC3 knockdown does not have a significant impact on HS-5 viability. **(b)** HDAC3 silencing in primary BMSC derived from RRMM is not cytotoxic. **(c)** and **(d)** HDAC3 knockdown does not impact HS-5 proliferation as measured using CFSE dilution assay and propidium iodide cell cycle analysis, respectively. **(e)** HDAC3 inhibition using BG45 is not cytotoxic towards HS-5 as measured by CCK-8 at 48 and 72H.

Figure S4

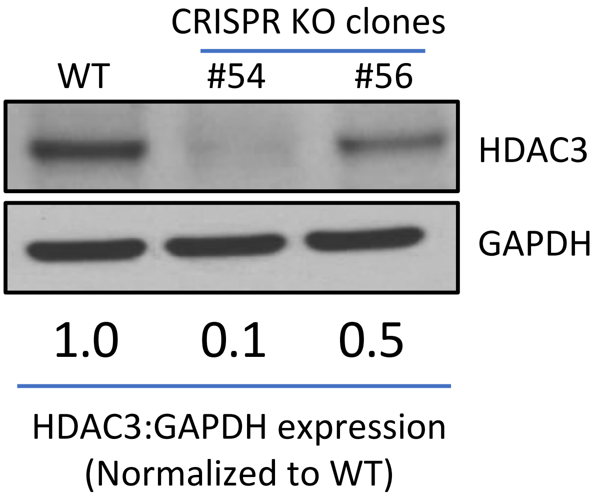

Figure S4: Western blot showing successful mono-allelic and bi-allelic CRISPR-Cas9 mediated KO of HDAC3.

Western blot showing successful mono-allelic and bi-allelic KO of HDAC3. Quantification was performed using ImageJ.

Figure S5

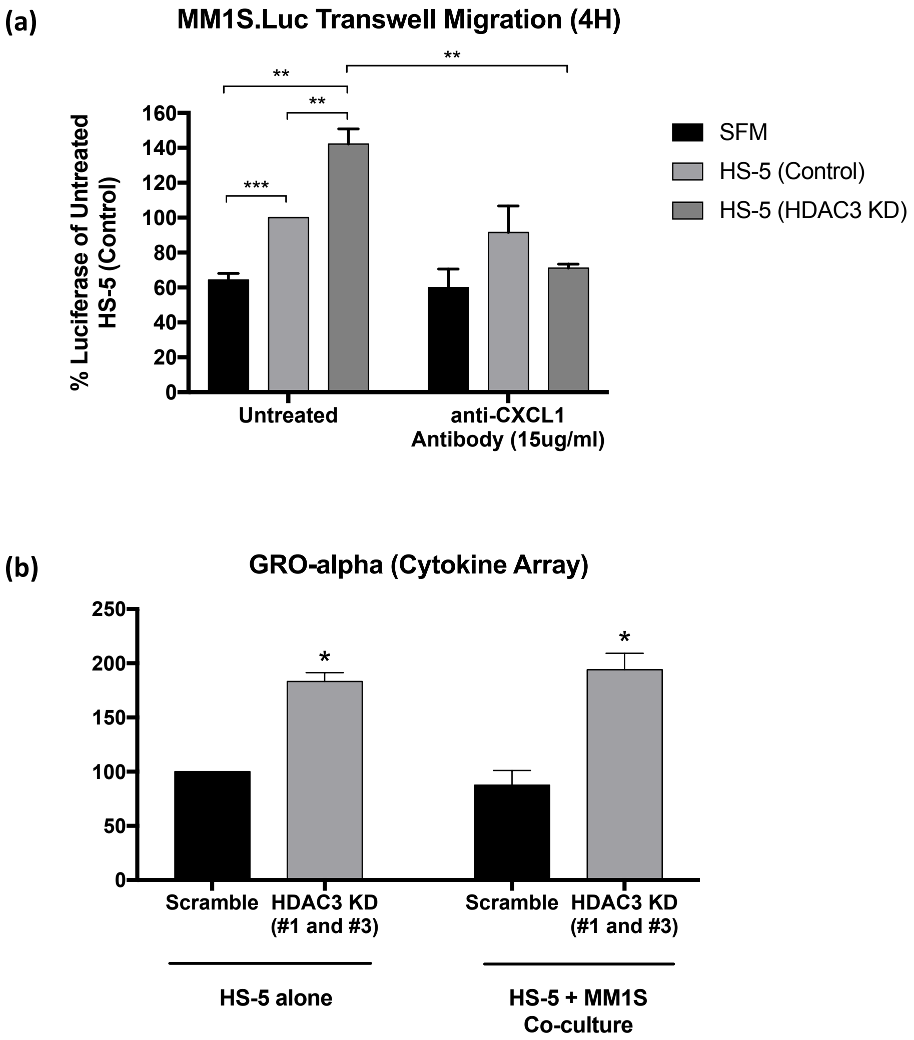

Figure S5: HDAC3 silencing in HS-5 results in significant increase in migration of MM cells

(a) Transwell migration studies were performed. Briefly, HS-5 was plated on the bottom chamber overnight while MM1S.Luc was seeded into the upper chamber (8µm pores; Costar). HDAC3 silencing in HS-5 led to an increase in MM chemotaxis which was inhibited by 15ug/ml of anti-CXCL1 antibody. **(b)** Cytokine array performed revealed a 1.9-fold increase in GRO-alpha (CXCL1) secreted when HDAC3 is silenced in HS-5 in the co-culture setting.

Figure S6

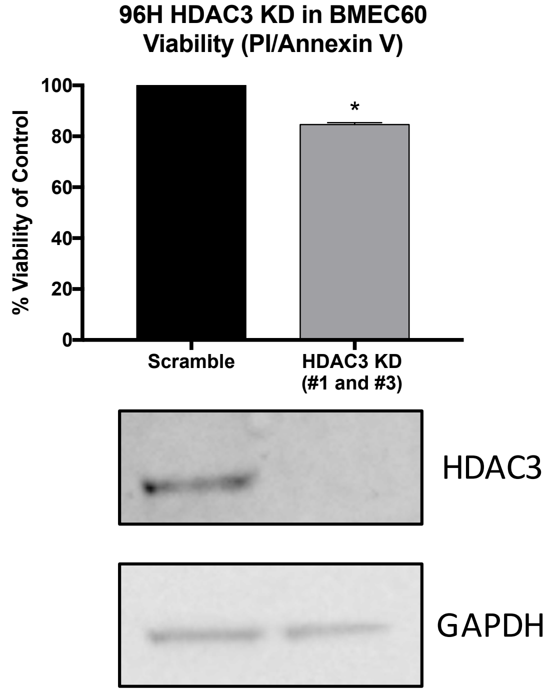

Figure S6: HDAC3 silencing in BMEC60 endothelial cell line resulted in a modest decrease in viability

HDAC3 silencing in BMEC60 endothelial cell line resulted in a modest decrease in viability as measured by PI/Annexin V flow cytometric analysis. Western blot showing knockdown of HDAC3 in BMEC60 at 96H.

Figure S7

(a)

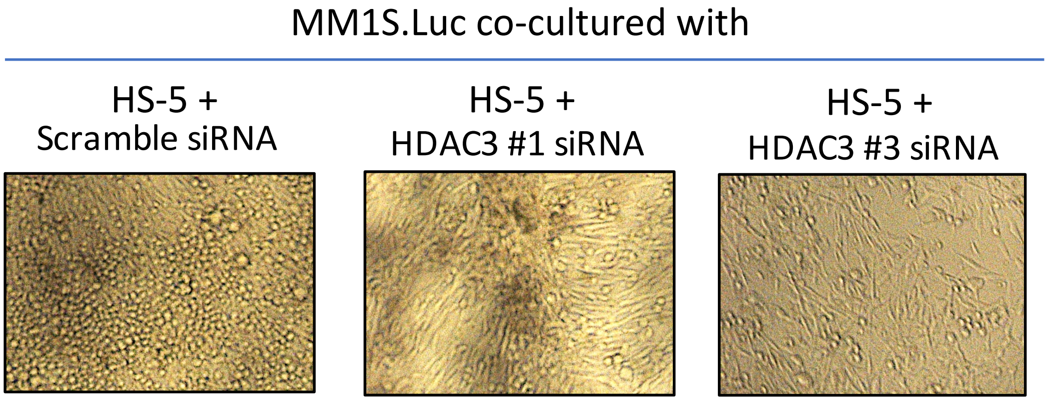

(b)

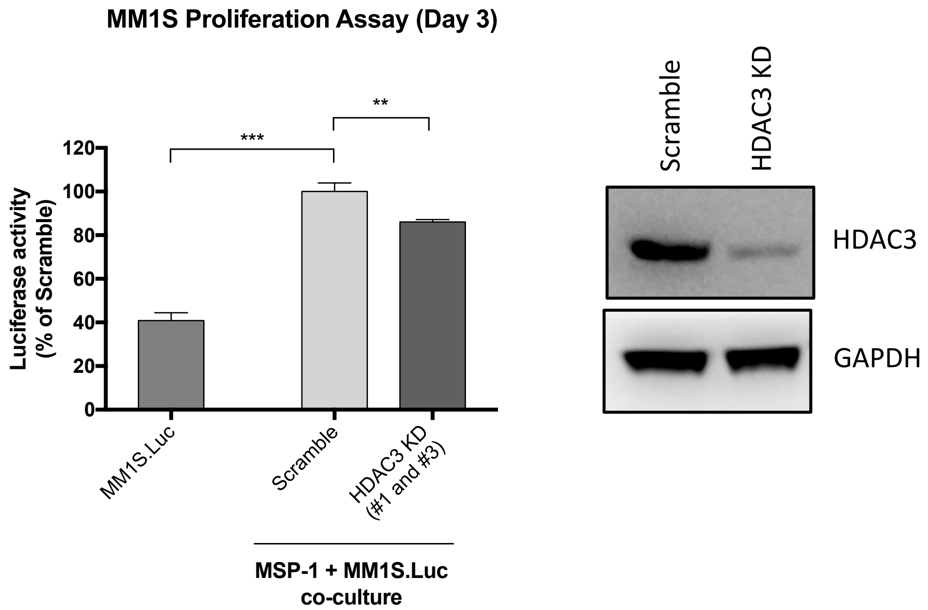

(c)

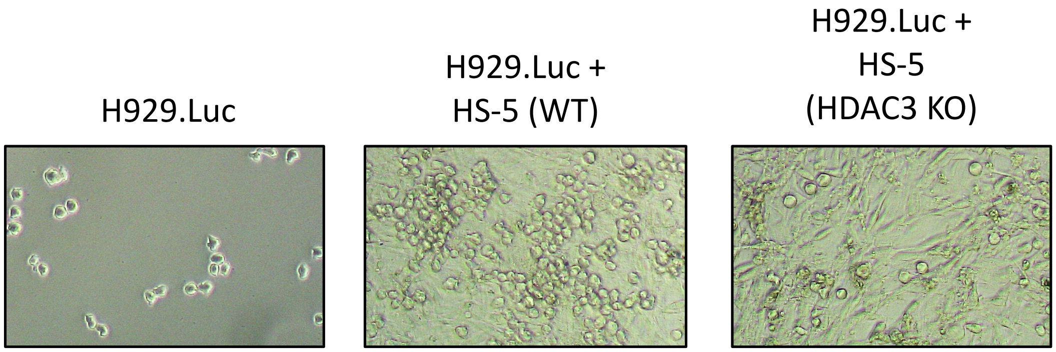

Figure S7: HDAC3 silencing in BMSC cell lines decreases MM proliferation

(a) HDAC3 siRNA knockdown in HS-5 significantly inhibits MM1S.Luc proliferation. Representative bright-field images of MM1S.Luc cells cultured with scrambled (left panel), HDAC3 #1 siRNA (middle panel), or HDAC3 #3 siRNA (right panel) HS-5. **(b)** HDAC3 knockdown in MSP-1 significantly inhibits MM1S.Luc proliferation (14% decrease in MM1S.Luc proliferation in HDAC3 KD, $p < 0.05$). **(c)** HDAC3 KO in HS-5 significantly inhibits H929.Luc proliferation. Representative bright-field images of H929.Luc cultured alone (left panel) or with HS-5 (WT) (middle panel) or HS-5 (HDAC3 KO) (right panel).

Figure S8

(a)

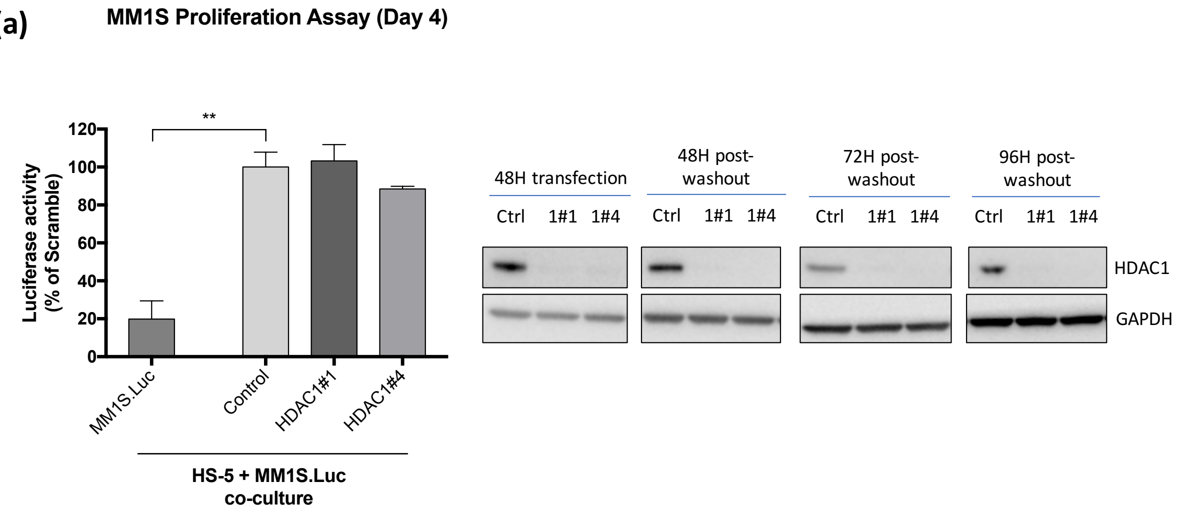

(b)

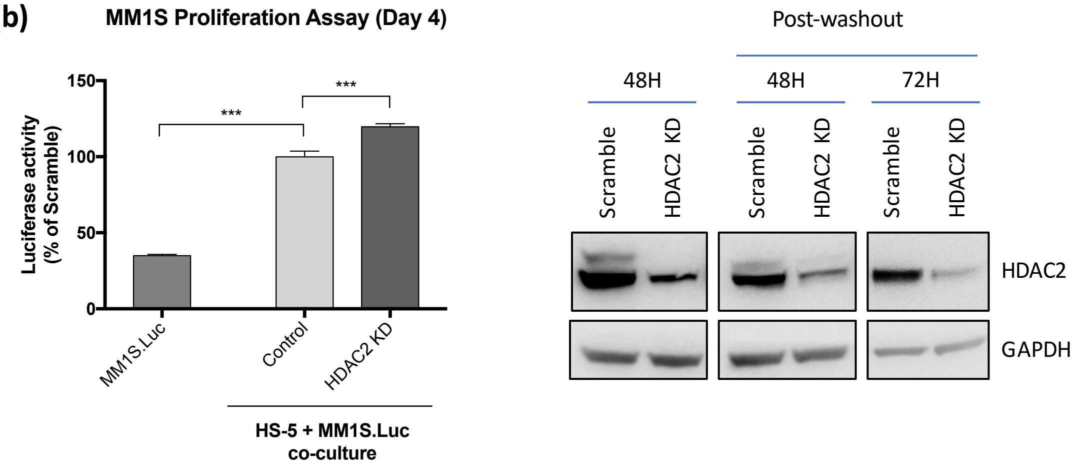

(c) **IC_{50} values of BG45 against the deacetylase activity of recombinant HDAC1-3 and HDAC6**

	HDAC1	HDAC2	HDAC3	HDAC6
**BG45**	2µM	2.2µM	289nM	>20µM

306

Figure S8: Experiments showing that treatment of HS-5 with 2µM BG45 results in MM growth inhibition due to on-target HDAC3 inhibition.

(a) HDAC1 knockdown in HS-5 does not affect MM proliferation in the co-culture setting. **(b)** HDAC2 knockdown in HS-5 increases MM proliferation in the co-culture setting. Taken together, these results suggest that the MM growth inhibitory effect of BG45-treated HS-5 is due to on-target HDAC3 inhibition. **(c)** Table showing the IC_{50} values of BG45 against HDAC1-3 and HDAC6. BG45 is a HDAC class-I inhibitor with selectivity for HDAC3 over HDAC1 and 2.

Figure S9

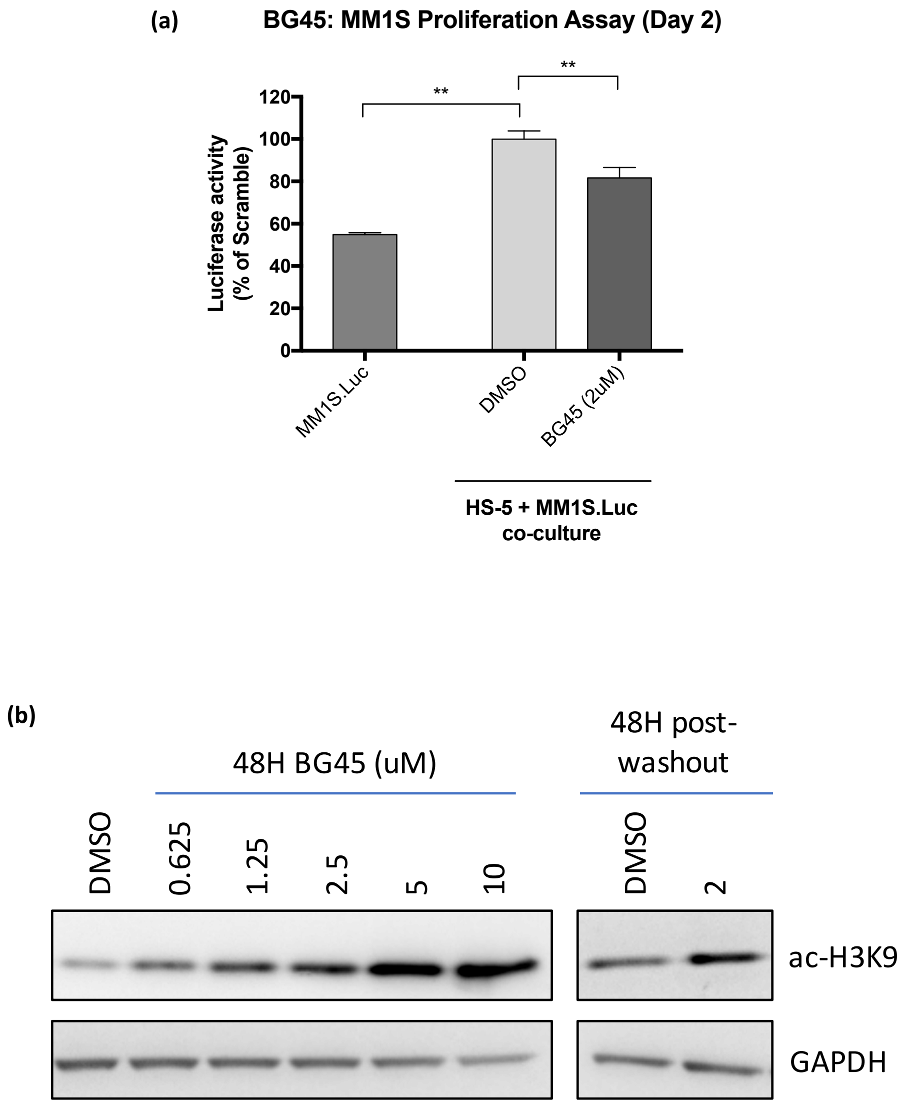

Figure S9: BG45 mediated HDAC3-selective inhibition in BMSCs result in decrease MM proliferation in co-culture.

(a) HDAC3 inhibition in HS-5 using 2µM BG45 led to a decrease in MM proliferation (18.3% decrease in MM proliferation in BG45 treated HS-5, $p < 0.05$). **(b)** Western blot showing that 2µM of BG45 inhibits HDAC3 in HS-5 as evidenced by increased acetylated-H3K9 HDAC3 inhibition that persisted for 48H post-washout of BG45.

Figure S10

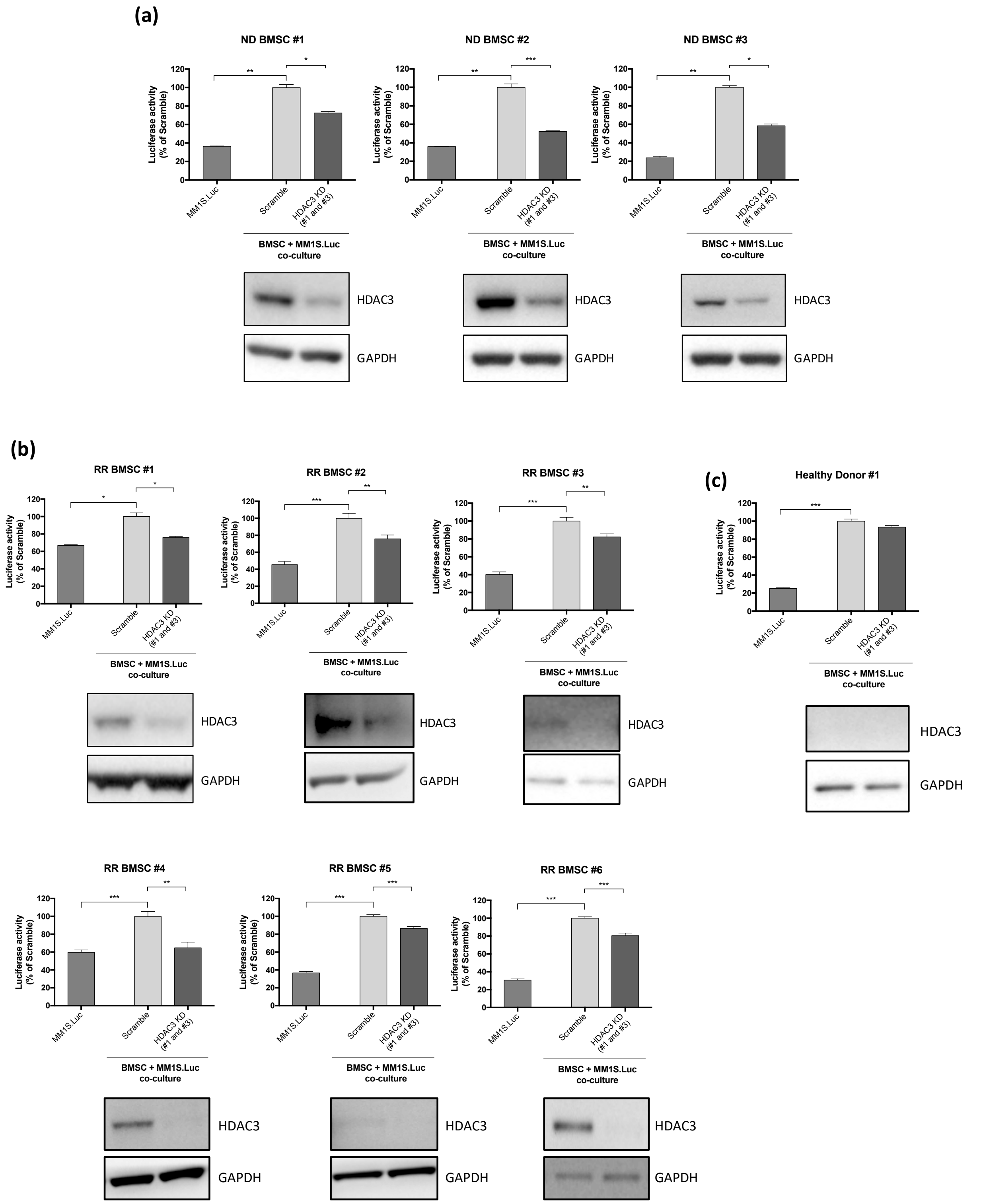
(a)
ND BMSC #1
ND BMSC #2
ND BMSC #3
Luciferase activity (% of Scramble)
MM1S.Luc
Scramble
HDAC3 KD (#1 and #3)
BMSC + MM1S.Luc co-culture
HDAC3
GAPDH
(b)
RR BMSC #1
RR BMSC #2
RR BMSC #3
(c)
Healthy Donor #1
RR BMSC #4
RR BMSC #5
RR BMSC #6

Figure S10: HDAC3 KD in primary human BM stromal cells derived from newly-diagnosed MM (ND BMSC), and refractory-relapsed MM (RR BMSC) trigger significant MM cell growth inhibition in MM-MSC co-culture setting

HDAC3 KD in **(a)** ND BMSC and **(b)** RR BMSC leads to significant decreases in MM1S.Luc proliferation (ND#1: 27.5% decrease in MM proliferation, $p < 0.05$; ND#2: 47.7% decrease in MM proliferation, $p < 0.05$; ND#3: 41.6% decrease in MM proliferation, $p < 0.05$); (RR#1: 24% decrease in MM proliferation, $p < 0.05$; RR#2: 24.2% decrease in MM proliferation, $p <0.05$; RR#3: 17.8% decrease in MM proliferation, $p < 0.05$; RR#4: 35.2% decrease in MM proliferation, $p < 0.05$; RR#5: 13.6% decrease in MM proliferation, $p < 0.05$; RR#6: 19.5% decrease in MM proliferation, $p < 0.05$). **(c)** HDAC3 KD in healthy donor-derived BMSC does not impact MM1S.Luc proliferation.

Figure S11

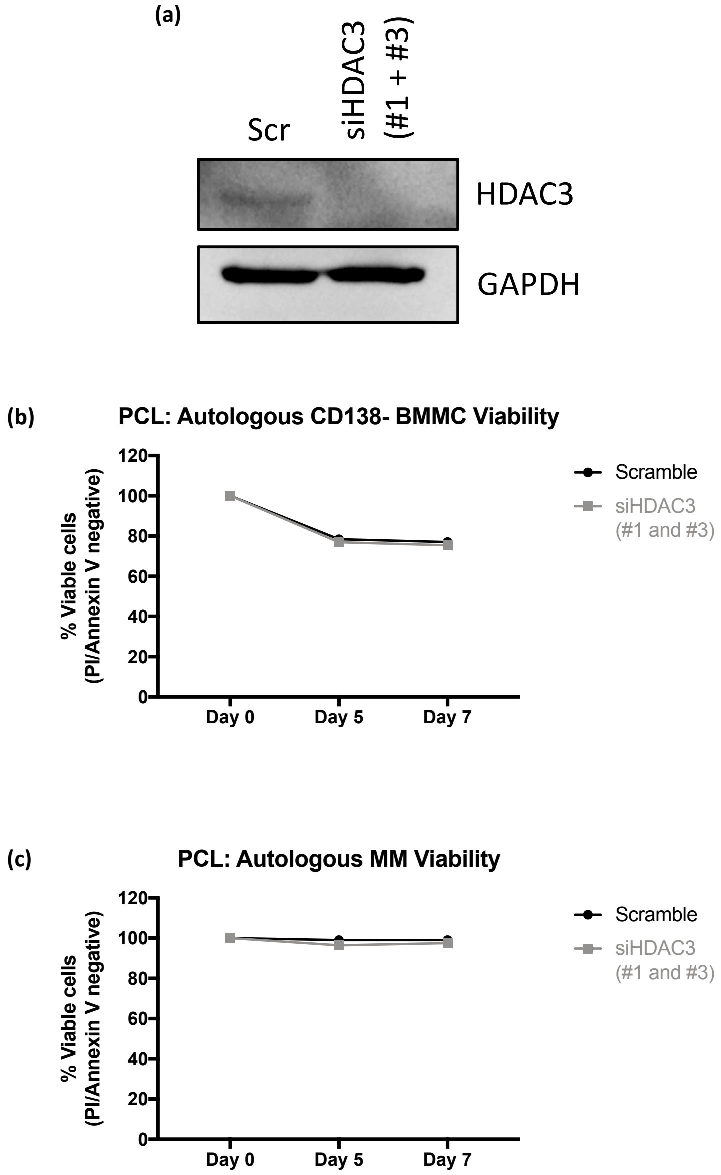

Figure S11: HDAC3 KD in CD138- BMMCs trigger significant autologous CD138+ MM cell growth inhibition in an autologous co-culture setting

(a) Western blot from CD138 negative BMMC lysate showing successful KD of HDAC3 in BMMC. **(b)** Chart showing viability over time of CD138 negative BMMC and **(c)** CD138 positive MM cells in scrambled and HDAC3 KD co-culture.

Figure S12

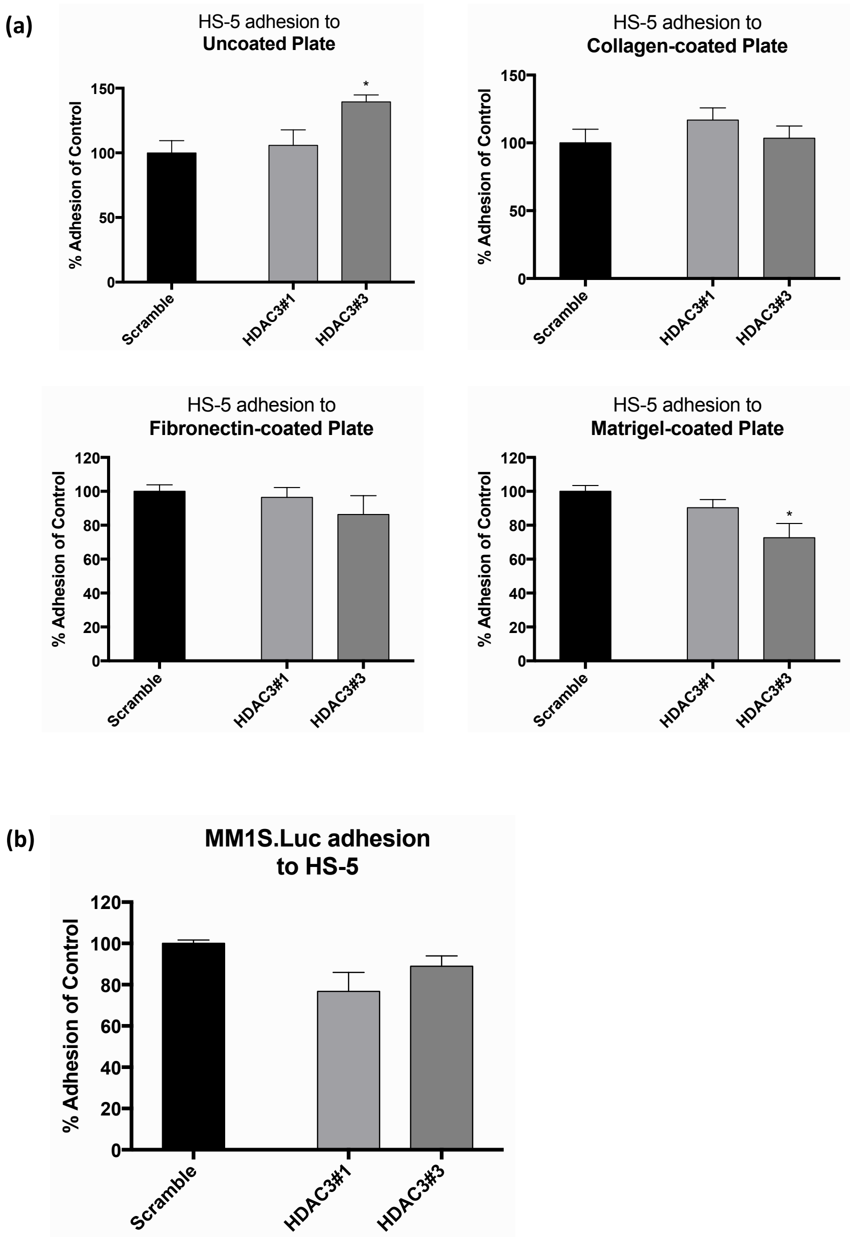

Figure S11: HDAC3 silencing in HS-5 does not impact HS-5 plate adhesion or MM1S.Luc adhesion

(a) HDAC3 siRNA knockdown does not impact HS-5 adhesion to uncoated, collagen, fibronectin, or matrigel-coated plate surfaces. **(b)** HDAC3 siRNA knockdown in HS-5 does not alter MM1S.Luc adhesion to HS-5.

Figure S13

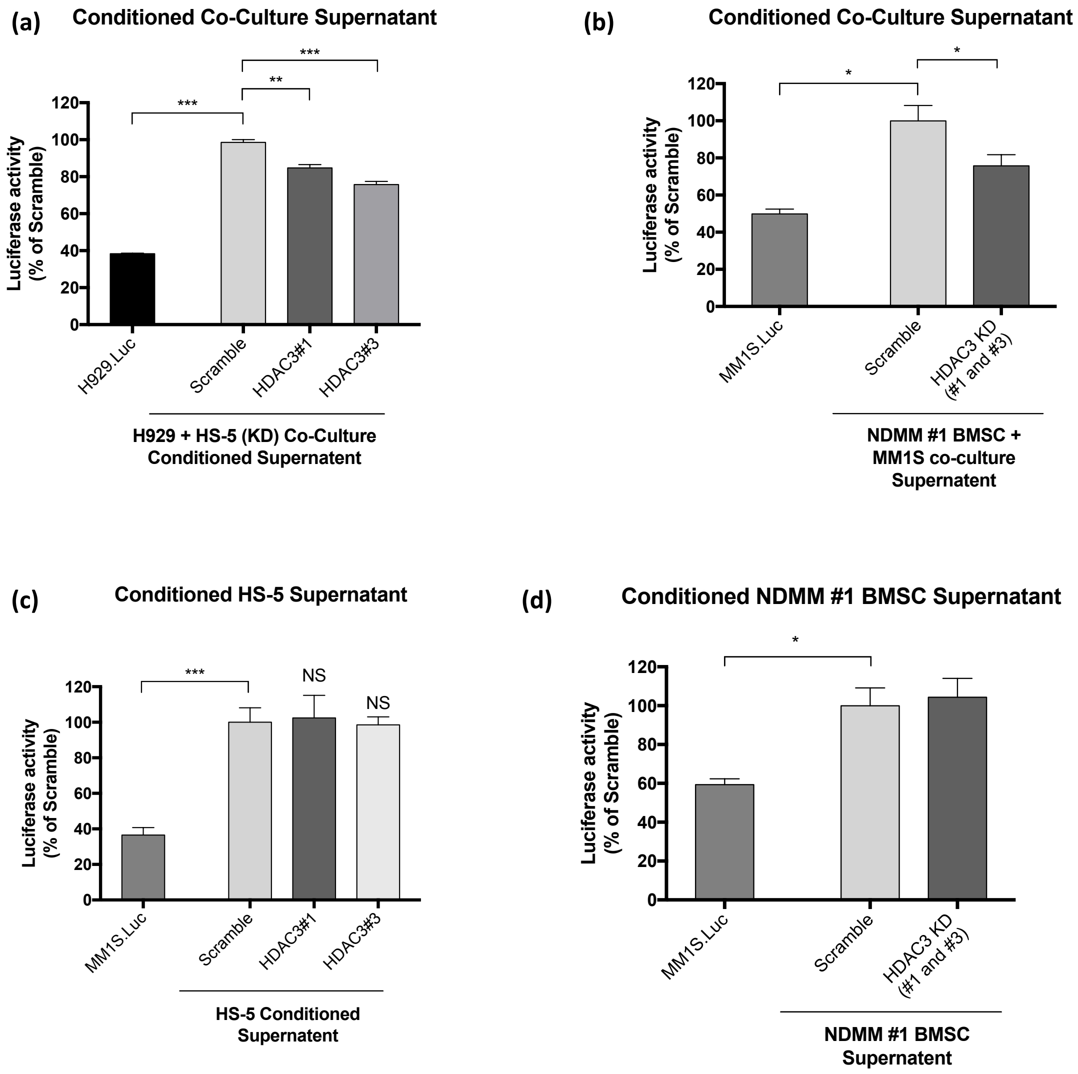

Figure S13: Paracrine-autocrine loop between HDAC3 KD BMSC and MM cells lead to inhibition of MM proliferation

(a) Conditioned media (CM) obtained from the co-culture of HDAC3 KD HS-5 and H929.Luc significantly inhibited H929.Luc proliferation. **(b)** CM obtained from the co-culture of HDAC3 KD NDMM-derived BMSC and MM1S.Luc significantly inhibited MM1S.Luc proliferation. **(c)** The CM from HDAC3 KD HS-5 alone does not have any anti-proliferative effect on MM1S.Luc. **(d)** CM from HDAC3 KD NDMM-derived BMSC cells alone failed to show an anti-proliferative effect.

Figure S14

(a)

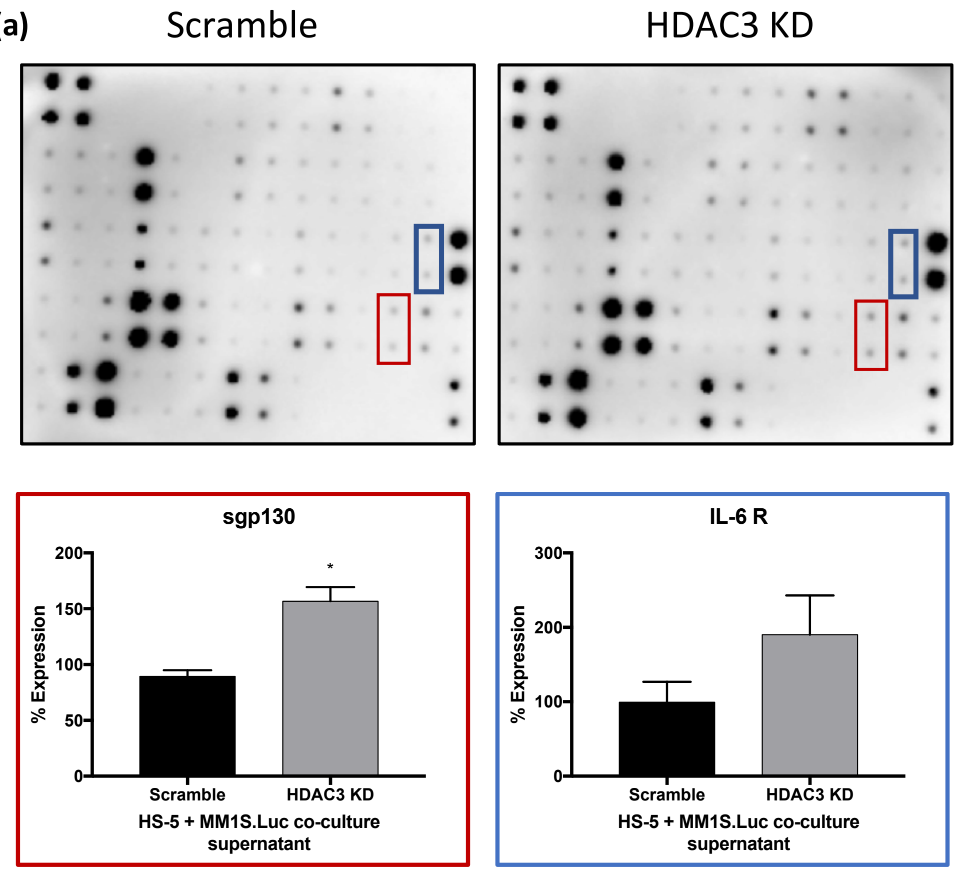

(b)	HS-5 + MM1S.Luc co-culture					
	Scramble (Fold Change)		HDAC3 KD (Fold change)		p value	Average Fold change of HDAC3KD vs Control
	N=1	N=2	N=1	N=2		
bFGF	1.1	1.2	2.5	3.3	0.044600	2.5
GRO-alpha	1.0	0.7	2.1	1.8	0.034692	1.9
sgp130	0.9	0.8	1.4	1.7	0.039560	1.8
IL-2 Ra	0.9	0.9	1.9	1.7	0.008508	1.8
sTNF RII	1.0	0.8	1.6	1.5	0.043514	1.7
Amphiregulin	1.0	1.0	1.6	1.7	0.003449	1.6
axl	1.5	1.3	2.1	2.4	0.033162	1.6
Acrp30	1.1	1.2	1.6	1.5	0.037029	1.6
Angiopoietin-2	1.1	1.1	1.6	1.4	0.037380	1.5
IL-1 R4/ST2	1.2	1.2	1.5	1.6	0.005805	1.3
IGFBP-2	1.0	0.9	1.4	1.3	0.023386	1.4
IGFBP-1	1.0	1.1	1.5	1.5	0.025169	1.4
VEGF-D	0.9	0.9	1.1	1.2	0.046389	1.3
I-TAC (CXCL11)	0.7	0.7	0.9	0.9	0.018209	1.2

Figure S14: Cytokine profiling of supernatant derived from HDAC3-silenced HS-5 stromal cells co-cultured with MM1S.Luc using RayBiotech C-series cytokine array.

(a) Cytokine profiling performed on supernatant derived from HS-5 (HDAC3 KD) + MM1S.Luc co-culture revealed a 1.8-fold increase in soluble-gp130 compared to control. **(b)** The table above shows the significant changes ($p < 0.05$; n=2 experiments) in cytokines released when HDAC3 was silenced in HS-5 cultured alone or with MM1S.Luc. Fold change was calculated by normalizing OD values to HS-5 (Scramble) alone.

Figure S15

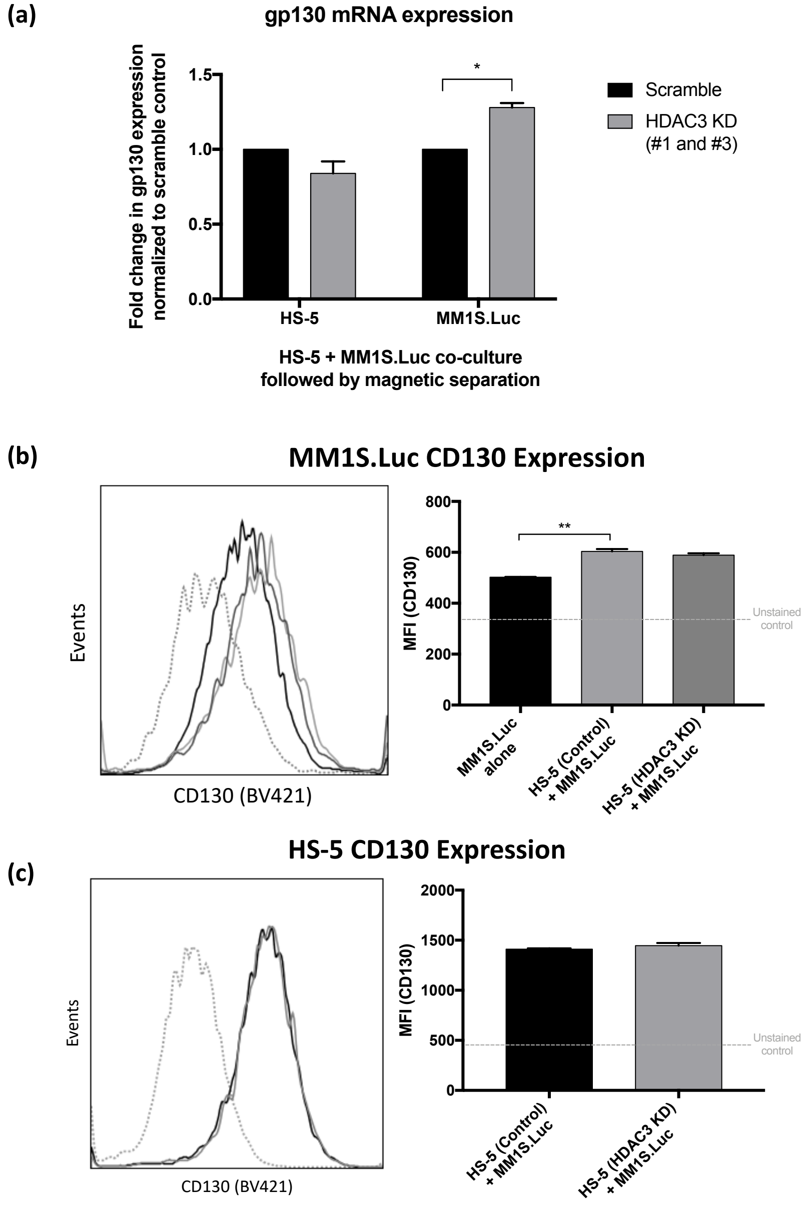

Figure S15: Conditioned supernatant from HDAC3 KD HS-5 plus MM1S.Luc co-culture triggers significant MM cell growth inhibition through the attenuation of IL-6 trans-signaling.

(a) RT-PCR performed on MM1S.Luc after magnetic separation from co-culture with HS-5 shows that HDAC3-silencing in HS-5 results in a 1.3-fold increase in MM1S.Luc gp130 mRNA expression. Flow cytometry analysis of cell surface gp130 shows that HDAC3-silencing in HS-5 does not alter surface expression of gp130 in both **(b)** MM1S.Luc and **(c)** HS-5.

Figure S16

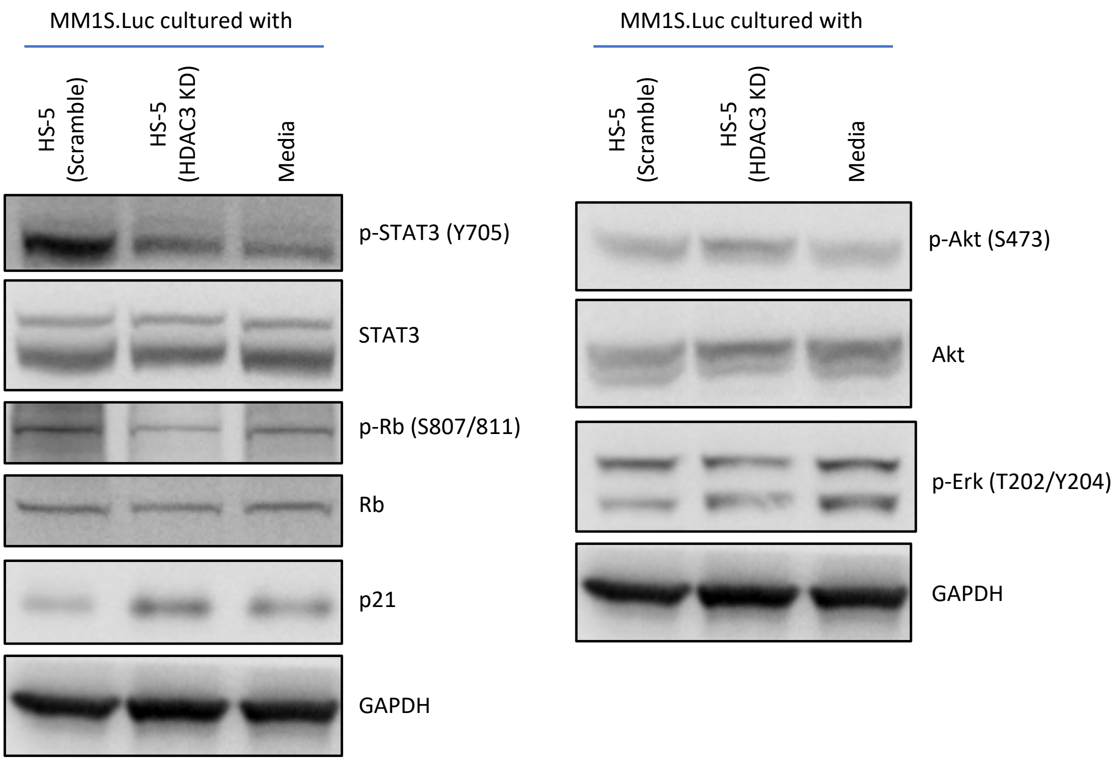

Figure S16: Western blot analysis showing changes in MM expression of key signaling proteins involved in the IL-6 trans-signaling pathway when HDAC3 was silenced in BMSCs

Western blot analysis showing that HDAC3 KD in HS-5 abrogates BMSC-induced STAT3 signaling in MM1S.Luc cells. No changes in ERK or Akt signaling were observed. GAPDH was used as loading control.

Figure S17

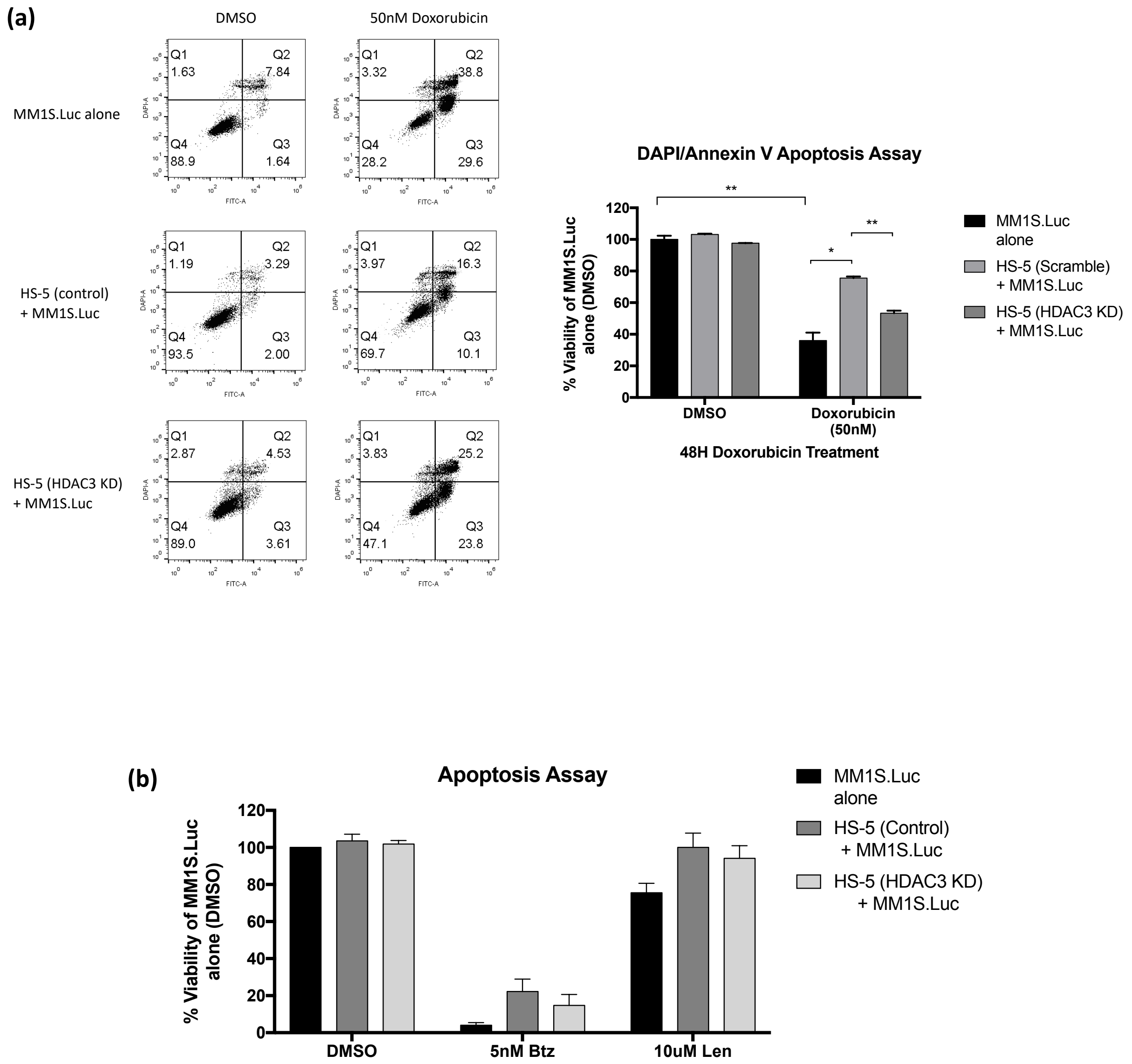

Figure S17: HDAC3 KD in HS-5 overcomes MM1S.Luc cell adhesion mediated-drug resistance (CAM-DR) to Doxorubicin

(a) HDAC3 silencing in HS-5 attenuated CAM-DR against doxorubicin. **(b)** HDAC3 silencing in HS-5 does not impact MM1S.Luc sensitivity to bortezomib or lenalidomide.

Figure S18

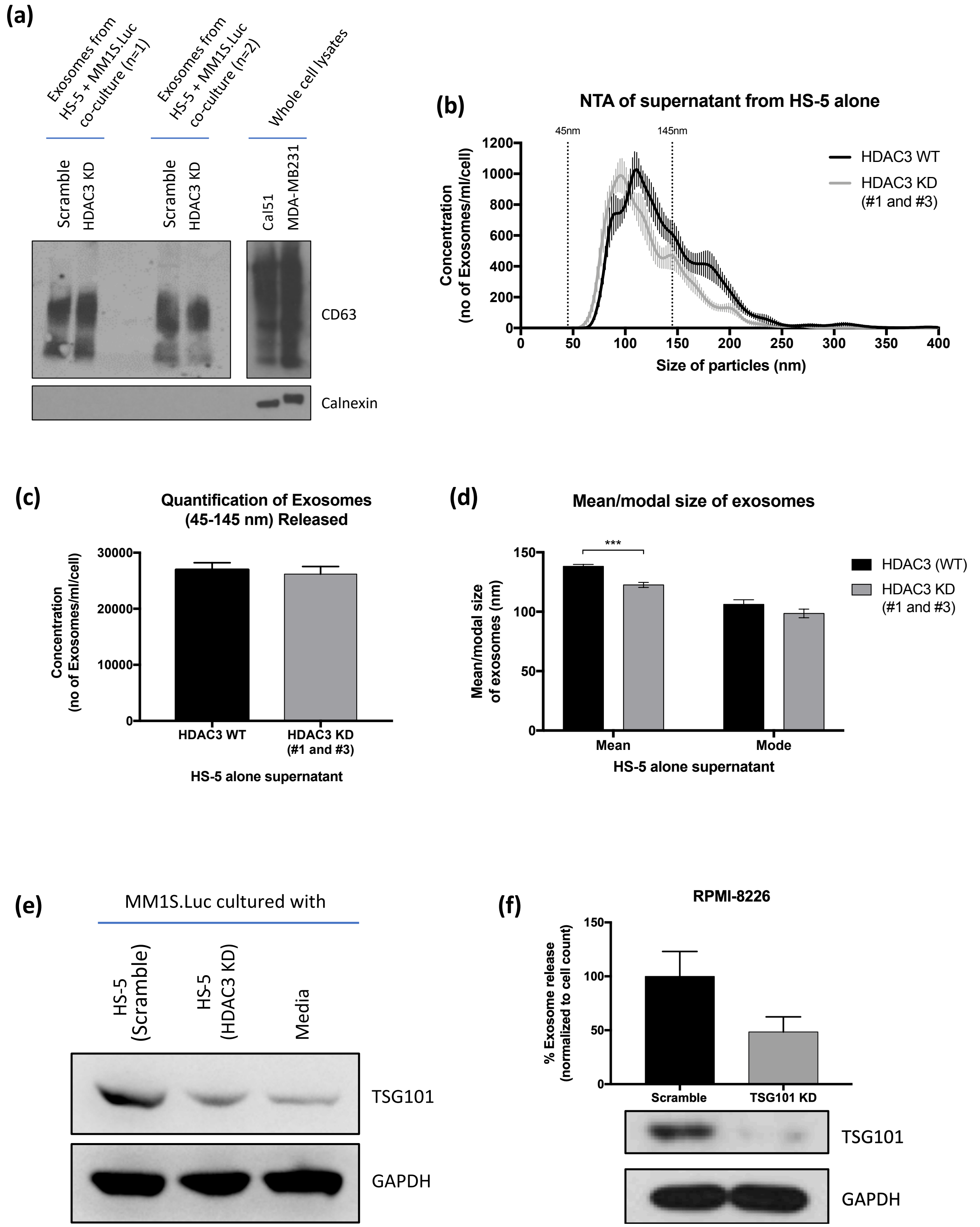
(a)
Exosomes from HS-5 + MM1S.Luc co-culture (n=1)
Exosomes from HS-5 + MM1S.Luc co-culture (n=2)
Whole cell lysates
Scramble
HDAC3 KD
Scramble
HDAC3 KD
Cal51
MDA-MB231
CD63
Calnexin
(b)
NTA of supernatant from HS-5 alone
45nm
145nm
HDAC3 WT
HDAC3 KD (#1 and #3)
Concentration (no of Exosomes/ml/cell)
Size of particles (nm)
(c)
Quantification of Exosomes (45-145 nm) Released
Concentration (no of Exosomes/ml/cell)
HDAC3 WT
HDAC3 KD (#1 and #3)
HS-5 alone supernatant
(d)
Mean/modal size of exosomes
***
Mean/modal size of exosomes (nm)
HDAC3 (WT)
HDAC3 KD (#1 and #3)
Mean
Mode
HS-5 alone supernatant
(e)
MM1S.Luc cultured with
HS-5 (Scramble)
HS-5 (HDAC3 KD)
Media
TSG101
GAPDH
(f)
RPMI-8226
% Exosome release (normalized to cell count)
Scramble
TSG101 KD
TSG101
GAPDH

Figure S18: Qualitative and quantitative changes in exosomes derived from HDAC3 KD HS-5 plus MM1S.Luc co-culture supernatant contributes to MM cell growth arrest

(a) Western blot analysis showing that the exosomes isolated from the conditioned media using Qiagen exoEasy kit were positive for exosomal marker CD63 but negative for calnexin. Whole cell lysates from Cal51 and MDA-MB231 serve as positive controls. **(b)** Nanoparticle tracking analysis (NTA) showing the mean/modal size and concentration of exosomes in the supernatant of HS-5 (HDAC3 KD) vs HS-5 (HDAC3 WT) cultured alone. **(c)** HDAC3 knockdown in HS-5 does not affect the quantity of exosomes secreted by HS-5 alone. **(d)** HDAC3 knockdown in HS-5 led to a small but significant decrease in the mean size of exosomes secreted by HS-5 without affecting the modal size of exosomes. **(e)** Western blot validation of reduced TSG101 expression in MM1S.Luc co-cultured with HDAC3-silenced HS-5. **(f)** siRNA knockdown of TSG101 in RPMI-8226 resulted in decreased exosome secretion as measured by NTA. Data was normalized to cell count for each condition.

Figure S19a

HS-5: Scramble siRNA HS-5 vs HDAC3 KD HS-5

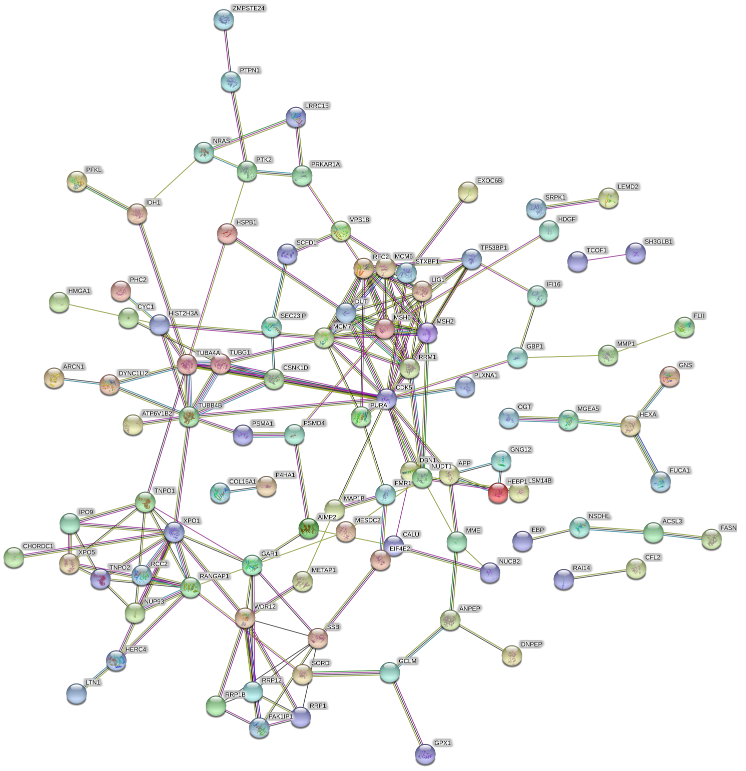

HDAC3 KD

Cellular protein localization	DNA unwinding	Mitotic cell cycle
ARCN1	HMGA1	CSNK1D
CDK5	MCM6	GIGYF2
CSNK1D	MCM7	IQGAP3
DBN1	PURA	LIG1
FAF1		MCM6
IPO9		MCM7
MESDC2		MSH2
MGEA5		NUP93
MYO1C		PSMA1
NRAS		PSMD4
NUP93		RANGAP1
RAB32		RCC2
RAB3B		RRM1
RAB5B		TUBA4A
RCC2		TUBB4B
SDCBP		TUBG1
SEC23IP		XPO1
SH3GLB1		
TNPO1		
TNPO2		
TOR1A		
VPS18		
XPO5		

	Biological Process	
Pathway ID	*Pathway description*	*FDR*
GO:0034613	cellular protein localization	0.001
GO:0006268	DNA unwinding involved in DNA replication	0.001
GO:0000278	mitotic cell cycle	0.007
GO:0006886	intracellular protein transport	0.007
GO:0006904	vesicle docking involved in exocytosis	0.007
GO:0006996	organelle organization	0.007
GO:0009892	negative regulation of metabolic process	0.007
GO:0032269	negative regulation of cellular protein metabolic process	0.007
GO:0044085	cellular component biogenesis	0.007
GO:0045184	establishment of protein localization	0.007
GO:0051246	regulation of protein metabolic process	0.007
GO:0051641	cellular localization	0.007
GO:0071840	cellular component organization or biogenesis	0.007
GO:0008104	protein localization	0.007
GO:0033036	macromolecule localization	0.007
GO:0051128	regulation of cellular component organization	0.007
GO:0010605	negative regulation of macromolecule metabolic process	0.011
GO:0015031	protein transport	0.014
GO:0032268	regulation of cellular protein metabolic process	0.014
GO:0032880	regulation of protein localization	0.014

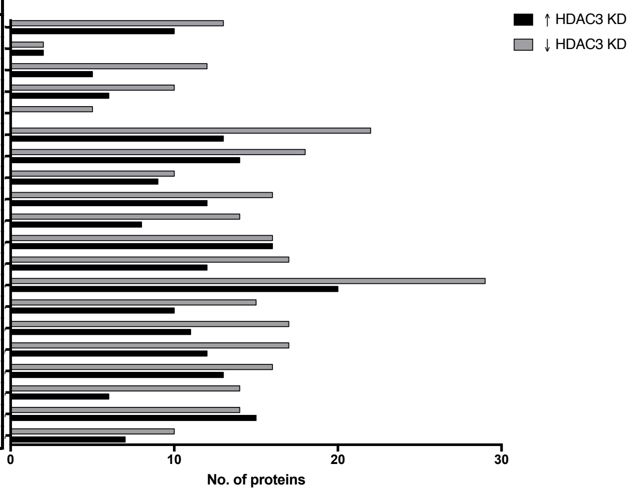

Figure S19b

HS-5: Scramble siRNA HS-5 + MM1S.Luc **vs**
HDAC3 KD HS-5 + MM1S.Luc

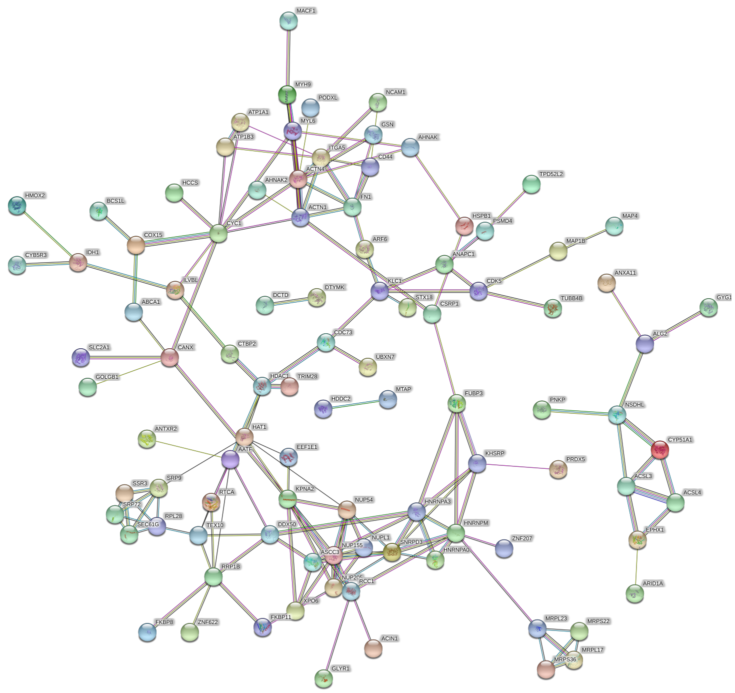

HDAC3 KD		
Cellular component disassembly	Complex subunit organization	Translatio n
ACIN1	ABCA1	EEF1E1
ARID1A	ACSL3	EIF1
CD44	ACTN4	MRPL17
FN1	AHNAK	MRPL23
GSN	ARID1A	MRPS22
KLC1	BCS1L	MRPS36
MRPL17	CDC73	RPL28
MRPL23	COX15	SEC61G
MRPS22	FAM103A1	SRP72
MRPS36	FN1	SRP9
NUP155	HAT1	SSR3
NUP205	HDAC1	
NUP54	HOOK3	
NUPL1	KLC1	
RPL28	MAP4	
	MRPL17	
	MRPL23	
	MRPS22	
	MRPS36	
	MYH9	
	NUP205	
	NUP54	
	NUPL1	
	RCC1	
	RPL28	
	SLC2A1	
	SNRPD3	
	TRIM28	
	TUBB4B	

Biological Process		
Pathway ID	*Pathway description*	*FDR*
GO:0022411	cellular component disassembly	0.00197
GO:0043933	macromolecular complex subunit organization	0.00833
GO:0006412	translation	0.00977
GO:0006518	peptide metabolic process	0.00977
GO:0007596	blood coagulation	0.00977
GO:0008152	metabolic process	0.00977
GO:0015031	protein transport	0.00977
GO:0051028	mRNA transport	0.00977
GO:0071822	protein complex subunit organization	0.00977
GO:0071840	cellular component organization or biogenesis	0.0105
GO:0016925	protein sumoylation	0.0132
GO:0010256	endomembrane system organization	0.0142
GO:0033036	macromolecule localization	0.0142
GO:0042060	wound healing	0.0142
GO:0044710	single-organism metabolic process	0.0142
GO:0006403	RNA localization	0.0164
GO:0016043	cellular component organization	0.0164
GO:0016482	cytoplasmic transport	0.0164
GO:0051641	cellular localization	0.0164
GO:1903923	positive regulation of protein processing in phagocytic vesicle	0.0164

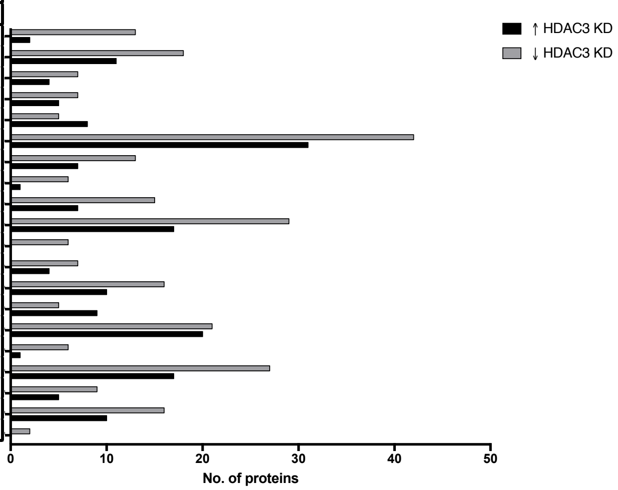

394

Figure S19c

MM1S.Luc: Scramble siRNA HS-5 + **MM1S.Luc** vs HDAC3 KD HS-5 + **MM1S.Luc**

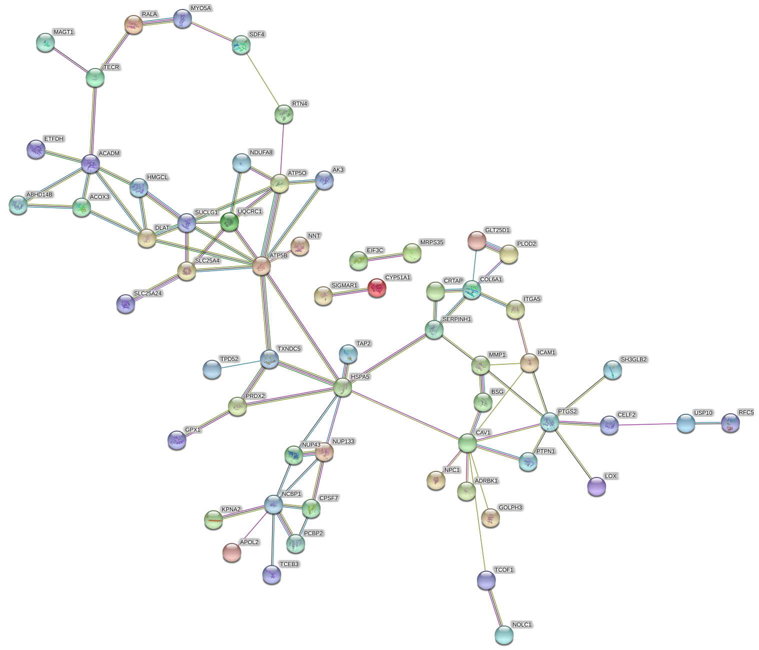

HDAC3 KD

Organism metabolic process	Redox process	Small mol. Metabolic process
ACADM	ACADM	ACADM
ADRBK1	ASPH	ACOX3
AK3	ATP5B	AK3
APOL2	ATP5O	APOL2
ASPH	CYP51A1	ATP5B
ATP5O	DHRS7	ATP5O
BSG	DLAT	BSG
CAV1	ETFDH	CAV1
COL6A1	GPX1	CRTAP
CRTAP	LOX	CYP51A1
CYP51A1	NDUFA8	ETFDH
DHRS7	NNT	GPX1
ETFDH	PLOD2	HDLBP
GPS1	PPOX	HMGCL
GPX1	PRDX2	LGMN
HDLBP	PTGS2	MYO5A
HMGCL	PYCR2	NDUFA8
HSPA5	SLC25A4	NNT
LGMN	SUCLG1	NPC1
LOX	TECR	NUP133
MAGT1		NUP43
MMP1		PCYT1A
MRPS35		PPOX
MYO5A		PTGS2
NDUFA8		PYCR2
NNT		SLC25A4
NPC1		TECR
NUP133		
NUP43		
PCYT1A		
PLOD2		
PPOX		
PRDX2		
PTGS2		
PTPN1		
PYCR2		
RFC5		
SERPINH1		
SLC25A4		
SUPV3L1		
TMEM165		
UBE2D3		
USP10		

Biological Process		
Pathway ID	*Pathway description*	*FDR*
GO:0044710	single-organism metabolic process	3.00E-05
GO:0055114	oxidation-reduction process	5.14E-05
GO:0044281	small molecule metabolic process	0.000805
GO:0044403	symbiosis, encompassing mutualism through parasitism	0.00472
GO:0016032	viral process	0.00574
GO:0006950	response to stress	0.022
GO:0008152	metabolic process	0.022
GO:0044237	cellular metabolic process	0.022
GO:0045333	cellular respiration	0.022
GO:0010033	response to organic substance	0.0271
GO:0044238	primary metabolic process	0.0271
GO:0071704	organic substance metabolic process	0.0271
GO:0030198	extracellular matrix organization	0.0286
GO:0019752	carboxylic acid metabolic process	0.0297
GO:0032787	monocarboxylic acid metabolic process	0.0297
GO:0040011	locomotion	0.0297
GO:0042060	wound healing	0.0297
GO:0044711	single-organism biosynthetic process	0.0297
GO:0006629	lipid metabolic process	0.0315
GO:0009060	aerobic respiration	0.0434

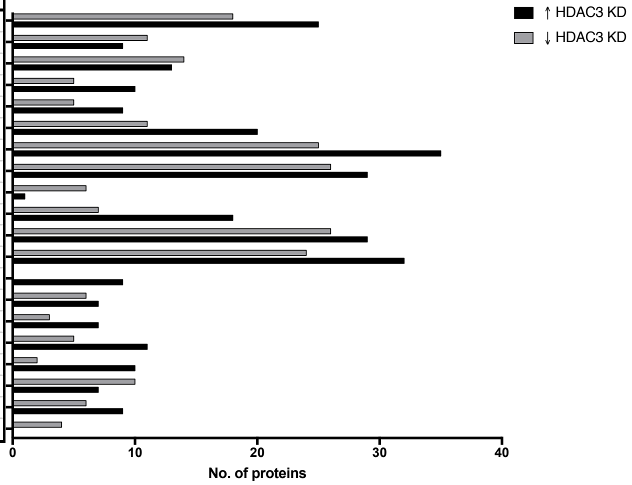

Figure S19: Mass-spectrometry analysis

Protein-protein interactions from the STRING interaction database. Proteins are represented as nodes. Disconnected nodes and low *p*-value interactions were filtered out. Proteins were also classified based on Gene Ontology (GO) biological processes. **(a)** Mass spectrometry analysis of scramble siRNA vs HDAC3 KD HS-5 cells cultured alone. **(b)** Mass spectrometry analysis of scramble siRNA vs HDAC3 KD HS-5 cells separated from the co-culture with MM1S.Luc. **(c)** Mass spectrometry analysis of MM1S.Luc cells separated from the co-culture with scramble siRNA vs HDAC3 KD HS-5 cells.

Figure S20

(a)

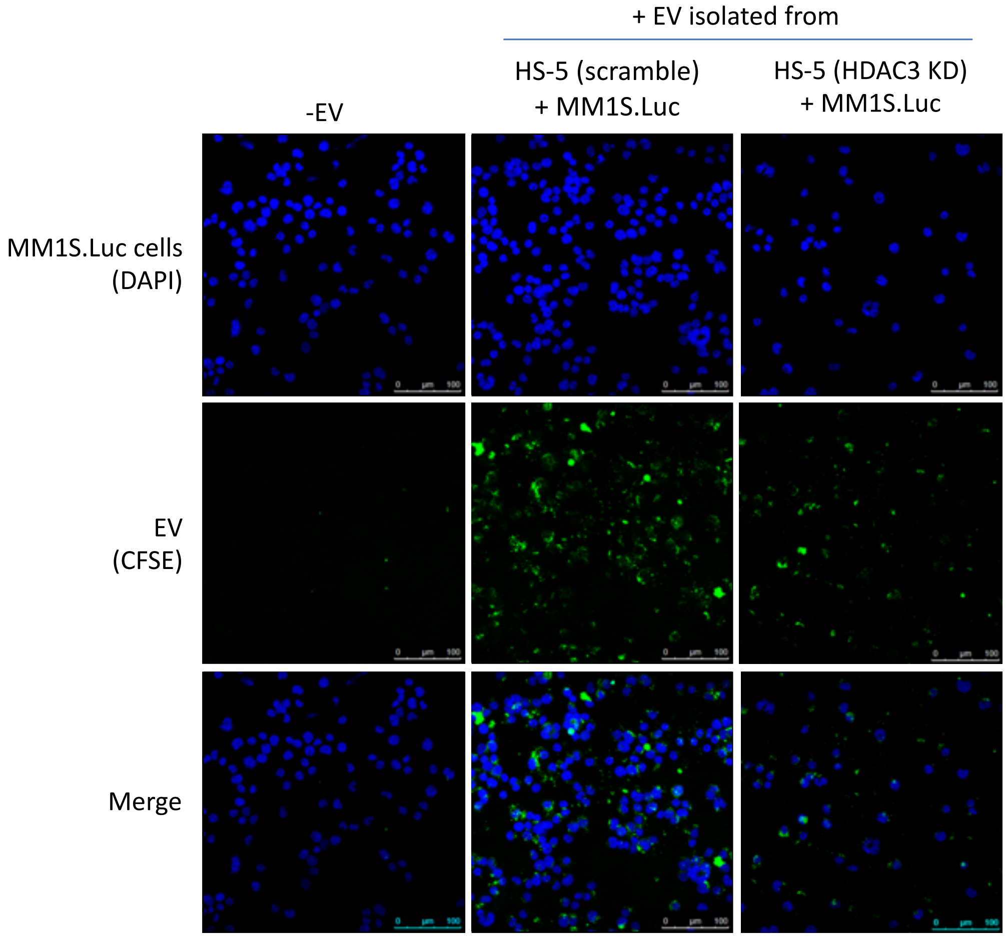

(b)

MM1S.Luc co-cultured with CFSE-labelled EV (6H)

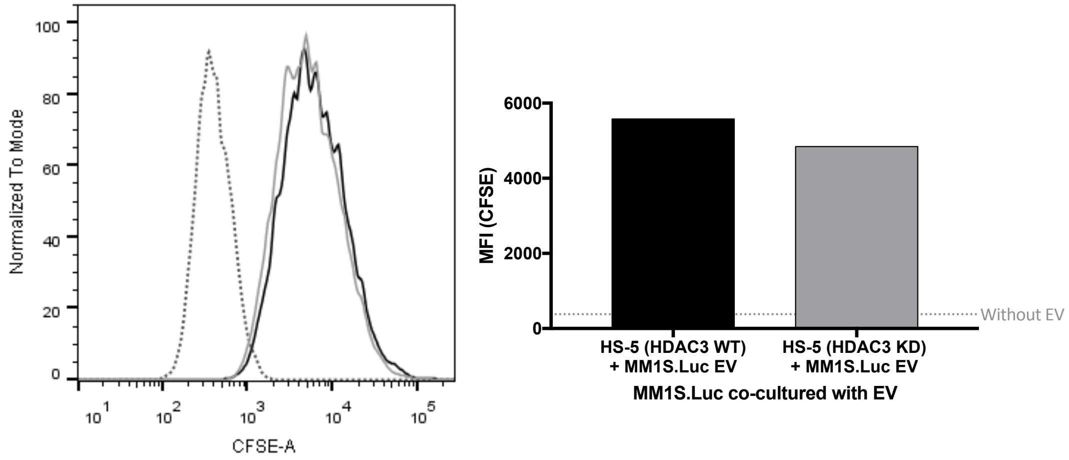

404

Figure S20: Confocal microscopy imaging and flow cytometric analysis showing uptake of exosomes into MM cells

(a) Confocal microscopy imaging showing the internalization of CFSE-labelled exosomes (green) into DAPI stained MM1S.Luc nuclei (blue). **(b)** Flow cytometric analysis showing uptake of CFSE-labelled exosomes into MM1S.Luc cells.

Figure S21

(a)

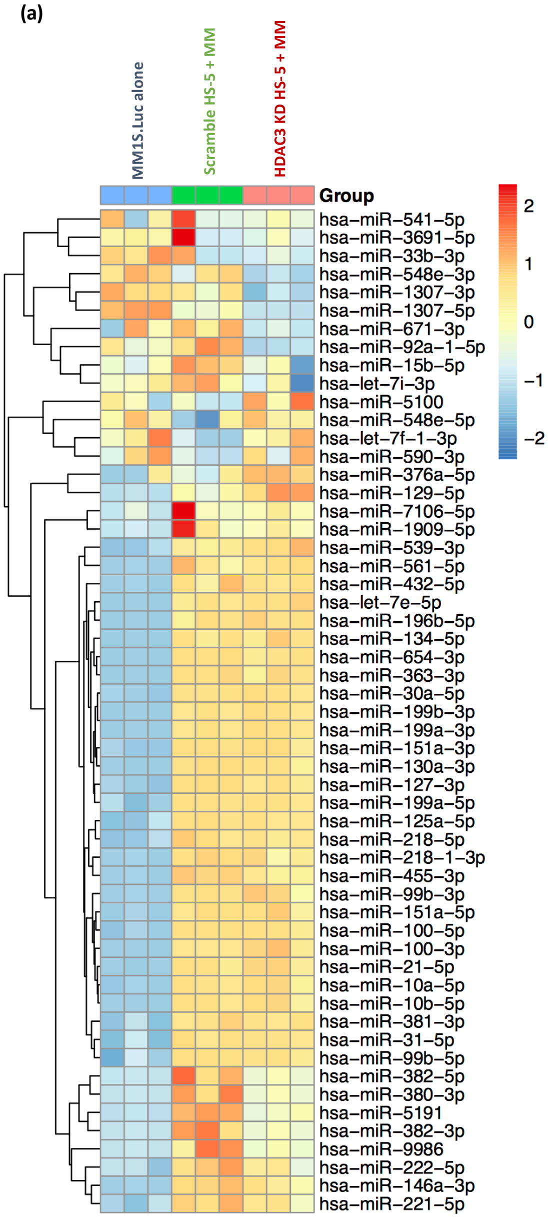

(b)

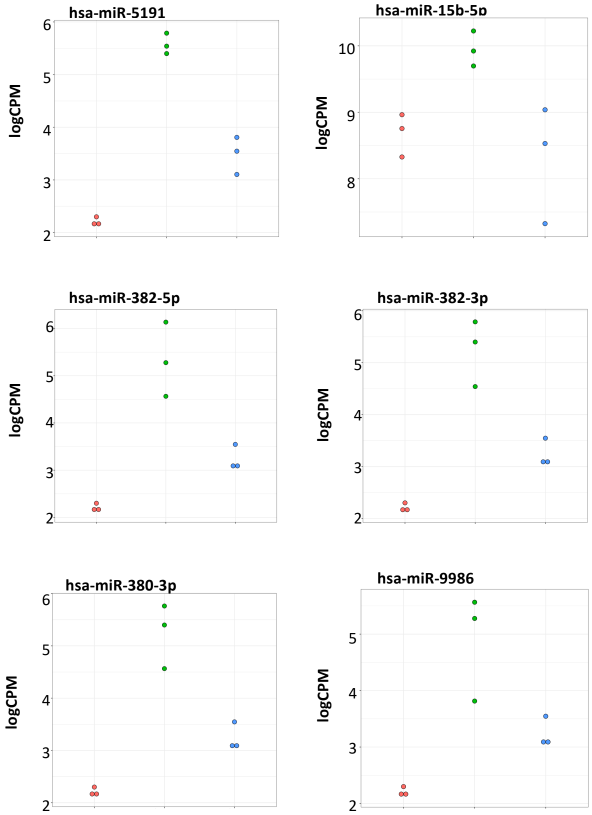

410

Figure S21: miRNA sequencing of exosomes isolated from the conditioned supernatant of MM1S.Luc alone, scramble siRNA HS-5 plus MM1S.Luc, and HDAC3 KD HS-5 plus MM1S.Luc

(a) Hierarchical clustering analysis for the identified differentially expressed small RNAs. The horizontal axis represents the exosomes derived from MM1S.Luc alone, scramble siRNA HS-5 + MM1S.Luc, and HDAC3 KD HS-5 + MM1S.Luc. These top genes were selected based on p-values. The z-scores of the logCPM are plotted. **(b)** Dot plots for miR380, miR382, miR15b, miR9986, and miR5191 show that these miRNAs are upregulated in exosomes derived from HS-5 (control) + MM1S.Luc when compared to MM1S.Luc alone and/or HS-5 (HDAC3 KD) + MM1S.Luc.
